# Does growing atmospheric CO_2_ explain increasing carbon sink in a boreal coniferous forest?

**DOI:** 10.1111/gcb.16117

**Published:** 2022-02-22

**Authors:** Samuli Launiainen, Gabriel G. Katul, Kersti Leppä, Pasi Kolari, Toprak Aslan, Tiia Grönholm, Lauri Korhonen, Ivan Mammarella, Timo Vesala

**Affiliations:** ^1^ Natural Resources Institute Finland Helsinki Finland; ^2^ Department of Civil and Environmental Engineering Duke University Durham North Carolina USA; ^3^ Faculty of Science Institute for Atmospheric and Earth System Research/Physics University of Helsinki Helsinki Finland; ^4^ Finnish Meteorological Institute Helsinki Finland; ^5^ University of Eastern Finland Joensuu Finland; ^6^ Faculty of Agriculture and Forestry Institute for Atmospheric and Earth System Research/Forest Sciences University of Helsinki Helsinki Finland; ^7^ Yugra State University Khanty‐Mansiysk Russia

**Keywords:** boreal forest, carbon and water fluxes, carbon balance, climate change, decadal trends, ecosystem modeling, eddy‐covariance, fluxnet, inter‐annual variability, leaf area index, water use efficiency

## Abstract

The terrestrial net ecosystem productivity (NEP) has increased during the past three decades, but the mechanisms responsible are still unclear. We analyzed 17 years (2001–2017) of eddy‐covariance measurements of NEP, evapotranspiration (ET) and light and water use efficiency from a boreal coniferous forest in Southern Finland for trends and inter‐annual variability (IAV). The forest was a mean annual carbon sink (252 [±42] gC $m^{‐2}$ $a^{‐1}$), and NEP increased at rate +6.4–7.0 gC $m^{‐2}$ $a^{‐1}$ (or ca. +2.5% $a^{‐1}$) during the period. This was attributed to the increasing gross‐primary productivity GPP and occurred without detectable change in ET. The start of annual carbon uptake period was advanced by 0.7 d $a^{‐1}$, and increase in GPP and NEP outside the main growing season contributed ca. one‐third and one‐fourth of the annual trend, respectively. Meteorological factors were responsible for the IAV of fluxes but did not explain the long‐term trends. The growing season GPP trend was strongest in ample light during the peak growing season. Using a multi‐layer ecosystem model, we showed that direct ${\mathrm{CO}}_{2}$ fertilization effect diminishes when moving from leaf to ecosystem, and only 30–40% of the observed ecosystem GPP increase could be attributed to ${\mathrm{CO}}_{2}$. The increasing trend in leaf‐area index (LAI), stimulated by forest thinning in 2002, was the main driver of the enhanced GPP and NEP of the mid‐rotation managed forest. It also compensated for the decrease of mean leaf stomatal conductance with increasing ${\mathrm{CO}}_{2}$ and LAI, explaining the apparent proportionality between observed GPP and ${\mathrm{CO}}_{2}$ trends. The results emphasize that attributing trends to their physical and physiological drivers is challenged by strong IAV, and uncertainty of LAI and species composition changes due to the dynamic flux footprint. The results enlighten the underlying mechanisms responsible for the increasing terrestrial carbon uptake in the boreal zone.

## Graphical abstract

Trends and inter‐annual variability of carbon and water exchange of a boreal coniferous‐dominated forest in southern Finland was studied by combining 17 years eddy‐covariance data with process‐model scenarios. We found increase of net carbon sink (+6.4–7.0 gC m^−2^ a^−1^) but no change in evapotranspiration. The increasing gross‐primary productivity was mostly attributed to management‐stimulated leaf‐area increase, not atmospheric CO_2_ fertilization. Meteorological conditions created strong inter‐annual variability but did not contribute to decadal trends.

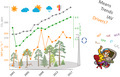

## 1 INTRODUCTION

The terrestrial carbon sink has increased during the past three decades partially offsetting the effect of increasing anthropogenic emissions on atmospheric ${\mathrm{CO}}_{2}$ concentration (Ahlström et al., 2015; Fu et al., 2017; Keenan et al., 2016; Schimel et al., 2001). However, the mechanisms responsible for enhancing net ecosystem productivity (NEP) are yet to be uncovered and continue to be the subject of debate (Keenan et al., 2016; Tharammal et al., 2019; Yue et al., 2015). At global and regional scales, the leading hypothesis associates increased NEP with rising atmospheric ${\mathrm{CO}}_{2}$ concentration ($c_{a}$) boosting gross‐primary productivity (GPP) directly and indirectly through increase in photosynthesizing biomass (Fernández‐Martínez et al., 2017; Fu et al., 2017; Schimel et al., 2015; Tharammal et al., 2019). Part of the increased global carbon sink has been also attributed to (i) hydrometeorological shifts such as rising spring and autumn air temperature and longer growing seasons, particularly at northern latitudes (Fu et al., 2017; Keenan et al., 2016; Piao et al., 2007), (ii) improved soil nutrient availability in response to increased atmospheric nitrogen deposition (de Vries et al., 2014; Magnani et al., 2007), (iii) increased diffuse light availability (Lee et al., 2018; Lucht et al., 2002; Mercado et al., 2009; Urban et al., 2007), and (iv) reduced ozone concentrations (Sitch et al., 2007). Clearly, these NEP boosting effects operate on varying timescales ranging from instantaneous to decadal (Stoy et al., 2009; Urbanski et al., 2007).

At the leaf scale, elevated $c_{a}$ increases the driving force for leaf photosynthesis (i.e., more ${\mathrm{CO}}_{2}$ molecules in the atmosphere leads to more collisions with the leaf surface, and more ${\mathrm{CO}}_{2}$ uptake per unit leaf area) but reduces stomatal conductance leading to increase in water use efficiency (WUE) (Ainsworth & Rogers, 2007; Cernusak et al., 2019). The exact magnitude of such direct ${\mathrm{CO}}_{2}$ fertilization effect is shown to vary across plant functional types, species and growth environments (Ainsworth & Rogers, 2007; McCarthy et al., 2010; Reid et al., 2003) and may differ between leaf and ecosystem scales (De Kauwe et al., 2013; Paschalis et al., 2017; Schäfer et al., 2002, 2003). In particular, growth of leaf‐area index (LAI), defined here as the half of the total area of plant leaves per unit ground area, has been shown to be one of the primary mechanism underlying global and regional increase of NEP (Haverd et al., 2020; Li et al., 2018). It also strongly regulates GPP and ecosystem respiration ($R_{e}$) in boreal and temperate forests (Launiainen et al., 2016; Lindroth et al., 2008; Wu et al., 2013). Changes in LAI also affect partitioning of evapotranspiration (ET) between physiologically controlled transpiration ($T_{r}$) and evaporative flux (E) (Launiainen et al., 2016; Leppä et al., 2020; Roberts, 1983), complicating interpretation of factors underlying variability and trends in WUE and GPP (Beer et al., 2009; Keenan et al., 2013; Knauer et al., 2018b).

The most coherent picture of mechanisms driving global and regional carbon sink and water use trends is based on Earth System Models and remote‐sensing inversions (Cernusak et al., 2019; Fu et al., 2017; Haverd et al., 2020; He et al., 2017; Hickler et al., 2008; Mastrotheodoros et al., 2017) that assimilate the findings from leaf‐scale studies, manipulation experiments (e.g., FACE, Ainsworth & Rogers, 2007; Leakey et al., 2009) and FluxNet observations (Baldocchi, 2020). As the longest eddy‐covariance (EC) time series date back to mid 90's and early 2000's, they are now starting to enable inter‐annual variability (IAV) and trends of ecosystem‐atmosphere carbon and water exchange to be detected directly from ecosystem level data (Baldocchi, 2020; Baldocchi et al., 2018). To date, synthesis studies have provided a diverse picture of the magnitude of decadal trends in boreal and temperate forests carbon and water exchange (Fernández‐Martínez et al., 2017; Keenan et al., 2013; Lavergne et al., 2019; Wang et al., 2018). Quite surprisingly, site‐level responses to changing environmental forcing have been analyzed in detail only in a few studies (Finzi et al., 2020; Grünwald & Bernhofer, 2007; Liu et al., 2019; Pilegaard & Ibrom, 2020; Pilegaard et al., 2011) motivating the work here.

The EC measurements of ${\mathrm{CO}}_{2}$ and water vapor fluxes above a boreal coniferous‐dominated forest in Hyytiälä, Southern Finland are examined with a lens on leaf and ecosystem gas exchange. The measurements were initiated in 1996 (Ilvesniemi et al., 2009; Markkanen et al., 2001) making its record one of the longest across FluxNet sites globally. The data shows increasing annual NEP as the positive trend in GPP exceeds that of $R_{e}$ (Fernández‐Martínez et al., 2017), prompting interest in:

- How much of the increased NEP and GPP is attributed to direct ${\mathrm{CO}}_{2}$ fertilization?
- Whether changes in climatic factors or in structure of the maturing forest can explain the increasing annual carbon sink?

These two questions are addressed by statistically analyzing nearly two decades of NEP and ET data. Explaining the long‐term trends and any causal links to drivers are then explored using a multilayer ecosystem model APES (Launiainen et al., 2015, 2016). The model offers a bridge between leaf‐ and ecosystem level gas exchange when hydroclimatic conditions along with structural and physiological adjustments are externally supplied. Thus, the model can discern “scale” issues between leaf and canopy responses to elevated atmospheric ${\mathrm{CO}}_{2}$ and how these are impacted by LAI changes occurring over much longer timescales than direct effects of hydroclimatic variability.

## 2 MATERIALS AND METHODS

### 2.1 Study site

The Hyytiälä SMEAR II station (Station for Measuring Ecosystem Atmosphere Relations) is located in Juupajoki, Southern Finland next to the Hyytiälä Forest Research Station established in 1910. The SMEAR II contributes to several monitoring programs including the European Integrated Carbon Observation System (ICOS) and FluxNet (site FI‐Hyy, 61° 51'N, 24° 17'E, 160–180m a.s.l.). It represents a coniferous dominated boreal forest on medium‐fertility mineral soils typical for the region. The average long‐term (1981–2010) annual air temperature ($T_{a}$) is $+3.5^{\circ }C$ and precipitation 711 mm.

The stand around the EC tower has been regenerated in 1962 by sowing by Scots pine seeds after clear‐cutting and prescribed burning. The soils are mainly mineral podzols formed after the last glaciation, whereas some bedrock outcrops with almost no topsoil are found close the EC tower. Small areas of shallow peat soils are located in depressions (Ilvesniemi et al., 2009). The dominant tree species are Scots pine (*Pinus sylvestris*), Norway spruce (*Picea abies*), and silver birch (*Betula pendula*). The ground vegetation consists of tree seedlings, dwarf shrubs (*Vaccinium sp*.) and mosses. Following normal silvicultural practices in even‐aged forestry, the first commercial thinning was done for part of the footprint in the winter of 2002 (Vesala et al., 2005).

#### 2.1.1 Eddy‐covariance and supplementary data

The EC measurements at SMEAR II commenced in the summer of 1996 (Markkanen et al., 2001). Every attempt was made to “harmonize” the analyzed data, which meant we discarded the pioneering years 1997–2000 when the measurement height was varied between 23 and 46 m and sampling lines for the gas analyzers were not heated. Likewise, data from 2018‐, after instrumentation were changed and measurement height increased, was discarded. Consequently, we focus on the 2001–2017 period during which the EC setup was located at 23 m from the mast base and included an ultrasonic anemometer (Solent Research 1012R2, Gill Instruments Ltd, Lymington, Hampshire, England) and a closed‐path infrared gas analyzer (LI‐6262, LI‐COR Biosciences, Lincoln, NE). For these 17 years, the 1/2 h NEP, ET, sensible heat and momentum fluxes were computed using the EddyUH software (Mammarella et al., 2016) following standard EC data processing (Aubinet et al., 2012) as detailed in Suppl. S1. The measured turbulent fluxes were corrected for storage changes below the measurement height using 1/2 h mean $T_{a}$ and gas mixing ratios sampled at several heights below the EC setup (Kolari et al., 2009; Launiainen, 2010).

Gaps in NEP were filled using REddyProc Online‐tool (Wutzler et al., 2018), which is the standard for FluxNet. The annual gap fractions and energy balance closure (EBC) are provided in Table 1. NEP was partitioned into GPP and $R_{e}$ using the nighttime‐approach (Reichstein et al., 2005) with both measured soil temperature ($T_{s}$) at 2 cm depth in mineral soil and $T_{a}$ as independent variables. To explore whether flux partitioning affects the study conclusions regarding annual balances and trends, we also applied a site‐specific method based on Kolari et al. (2009) and a daytime approach (Lasslop et al., 2010) for comparison (see Suppl. S2). Shorter than 2 h gaps in ET were linearly interpolated and longer gaps filled by common statistical gap‐filling algorithms (Reichstein et al., 2005). The EC source area (defined here as 80% of the flux footprint) was estimated for each year using measured sensible heat flux, friction velocity ($u_{*}$), and turbulent velocity variances externally supplied to a standard source‐weight function model (Kljun et al., 2015).

**TABLE 1:** Annual values

	2001	2002	2003	2004	2005	2006	2007	2008	2009	2010	2011	2012	2013	2014	2015	2016	2017	Mean (±SD)
Annual																		
${\mathrm{NEE}}_{\mathrm{Ts}}$ (g $m^{‐2}$)	180	236	171	221	238	204	239	249	312	240	288	268	273	287	306	285	284	252 (42)
${\mathrm{NEE}}_{\mathrm{SP}}$ (g $m^{‐2}$)	165	209	137	210	216	186	236	239	305	227	287	272	268	274	297	270	274	239 (48)
${\mathrm{GPP}}_{\mathrm{Ts}}$ (g $m^{‐2}$)	1013	1069	1158	1117	1146	1040	1171	1070	1228	1135	1257	1180	1249	1167	1166	1224	1122	1148 (71)
${\mathrm{GPP}}_{\mathrm{Ta}}$ (g $m^{‐2}$)	1044	1105	1183	1148	1182	1078	1203	1106	1268	1169	1288	1209	1273	1206	1199	1258	1148	1180 (70)
${\mathrm{GPP}}_{\mathrm{SP}}$ (g $m^{‐2}$)	1059	1119	1043	1153	1131	1072	1178	1098	1202	1140	1265	1213	1199	1161	1182	1201	1104	1148 (61)
${\mathrm{GPP}}_{\mathrm{DT}}$ (g $m^{‐2}$)	1003	1034	1008	1107	1105	1048	1112	1088	1176	1112	1252	1166	1130	1166	1172	1179	1137	1117 (67)
$R_{e,Ts}$ (g $m^{‐2}$)	833	833	986	896	908	836	932	821	915	895	968	912	975	880	861	939	838	896 (53)
$R_{e,Ta}$ (g $m^{‐2}$)	865	869	1011	927	943	874	964	857	956	929	1000	941	999	919	893	973	864	928 (52)
$R_{e,SP}$ (g $m^{‐2}$)	894	911	906	943	916	886	942	859	897	913	978	941	930	887	885	930	831	909 (35)
$R_{e,DT}$ (g $m^{‐2}$)	766	725	807	823	805	803	813	798	807	807	916	805	756	848	837	844	796	809 (41)
ET (mm)	356	345	325	302	296	330	377	366	372	394	340	330	352	357	368	371	337	348 (26)
$T_{a}$ (°C)	3.9	4.3	4.1	4.0	4.5	4.8	4.7	4.9	3.8	2.5	5.2	3.2	5.1	5.2	5.7	4.5	4.3	4.4 (0.8)
$T_{s}$ (°C)	5.3	6.7	5.9	5.1	5.6	5.6	5.8	5.5	5.1	5.4	6.0	5.5	5.9	6.0	6.2	5.7	5.1	5.7 (0.4)
D (kPa)	0.25	0.31	0.25	0.27	0.30	0.33	0.26	0.23	0.26	0.28	0.28	0.20	0.26	0.28	0.23	0.23	0.19	0.26 (0.04)
Par (${\mu \mathrm{molm}}^{‐2}$ s^−1^)	200.4	220.9	199.7	194.9	215.6	223.9	209.7	185.0	200.7	197.7	197.5	186.7	203.0	195.1	192.2	187.8	202.7	200.8 (11.2)
$c_{a}$ (ppm)	374.7	374.8	376.2	378.4	378.6	379.6	386.9	387.4	391.6	393.5	395.3	398.6	400.1	401.5	403.5	406.8	409.5	390.4 (11.8)
GS start (doy)	118	116	130	112	129	119	89	120	119	137	117	133	132	120	127	124	142	123 (12)
GS end (doy)	298	268	291	289	301	307	288	308	287	290	319	300	295	294	284	288	297	294 (11)
GS length (days)	180	152	161	177	172	188	199	188	168	153	202	167	163	174	157	164	155	172 (15)
SCU (doy)	101	104	110	106	97	108	82	97	102	101	105	111	112	67	91	90	78	98 (12)
ECU (doy)	254	292	239	247	269	273	268	270	289	257	256	267	266	272	267	278	262	266 (13)
CUP (days)	153	188	129	142	172	165	186	173	187	157	152	156	154	205	177	189	183	169 (20)
NEP gaps (%)	33.9	35.8	41.8	31.9	26.7	31.0	29.8	25.2	33.4	41.5	34.7	35.5	37.4	29.3	27.2	45.3	31.5	33.6 (5.6)
EBC (−)	0.90	0.81	0.83	0.82	0.78	0.84	0.84	0.81	0.84	0.86	0.83			0.90	0.92	0.89	0.90	0.85 (0.04)
May–September																		
${\mathrm{NEE}}_{\mathrm{Ts}}$ (g $m^{‐2}$)	280	307	268	270	288	320	305	325	394	294	379	348	379	329	355	324	353	325 (39)
${\mathrm{NE}}_{\mathrm{SP}}$ (g $m^{‐2}$)	272	285	240	264	289	309	304	319	383	291	377	349	377	322	348	309	351	317 (42)
${\mathrm{GPP}}_{\mathrm{Ts}}$ (g $m^{‐2}$)	903	953	1044	966	1007	927	1002	923	1106	1014	1095	1064	1119	996	982	1064	965	1008 (66)
${\mathrm{GPP}}_{\mathrm{Ta}}$ (g $m^{‐2}$)	936	989	1070	995	1041	963	1036	956	1146	1051	1129	1097	1145	1031	1014	1094	993	1040 (66)
${\mathrm{GPP}}_{\mathrm{SP}}$ (g $m^{‐2}$)	949	998	941	998	991	963	1016	951	1085	1010	1108	1090	1082	991	1000	1038	967	1011 (53)
${\mathrm{GPP}}_{\mathrm{DT}}$ (g $m^{‐2}$)	893	903	889	932	952	925	941	934	1053	972	1084	1022	1013	991	992	1017	971	970 (56)
$R_{e,Ts}$ (g $m^{‐2}$)	623	646	776	696	719	607	696	598	712	721	716	716	740	667	627	740	611	683 (55)
$R_{e,Ta}$ (g $m^{‐2}$)	656	682	802	725	753	643	730	631	752	757	750	749	766	702	659	770	640	716 (54)
$R_{e,SP}$ (g m^−2^)	678	713	701	734	702	654	712	632	701	720	731	741	705	669	652	730	616	694 (37)
$R_{e,DT}$ (g $m^{‐2}$)	608	582	623	651	651	606	623	607	659	667	726	640	603	666	636	695	605	638 (37)
ET (mm)	305	298	288	255	250	261	289	263	303	328	276	268	294	299	289	291	241	282 (23)
LUE (mmol ${\mathrm{mol}}^{‐1}$)	15.89	15.18	18	19.14	17.09	14.4	17.24	17.49	19.05	17.84	19.73	20.21	19.62	18.08	17.81	19.42	16.88	17.83 (1.63)
WUE (mmol ${\mathrm{mol}}^{‐1}$)	4.44	4.71	5.12	5.61	5.97	5.33	5.22	5.35	5.44	4.66	5.83	6	5.58	4.93	5.07	5.47	5.99	5.34 (0.48)
$T_{a}$ (°C)	12.9	14.2	13.4	12.2	12.9	14.3	12.8	11.5	13.0	13.9	14.0	12.2	14.1	13.6	12.5	13.7	11.4	13.1 (0.9)
$T_{s}$ (°C)	9.7	11.1	10.3	9.8	10.2	10.3	10.5	9.8	10.2	11.0	11.2	10.3	11.2	11.2	10.5	11.2	9.2	10.4 (0.6)
D (kPa)	0.72	0.81	0.67	0.69	0.84	0.95	0.76	0.7	0.77	0.9	0.87	0.68	0.79	0.88	0.7	0.72	0.61	0.77 (0.09)
PAR (μ mol $m^{‐2}\;s^{‐1}$)	353.8	384.5	340.7	312.6	363.3	400.6	359.9	330.2	355.9	350.3	340.0	327.8	347.7	340.5	343.3	335.2	355.9	349.5 (20.8)
P (mm)	389	282	304	411	414	210	319	482	325	437	479	482	318	410	334	496	425	383 (83)
$G_{s}$ (mol $m^{‐2}\;s^{‐1}$)	0.20	0.17	0.20	0.19	0.15	0.13	0.18	0.18	0.19	0.18	0.16	0.20	0.18	0.17	0.20	0.20	0.18	0.18 (0.02)

The following meteorological variables measured at 17 and 33 m above the ground were used in the analysis and model scenarios: net radiation ($R_{n}$), direct and diffuse global ($R_{g}$), photosynthetically active (PAR) and near‐infra red (NIR) radiation, $T_{a}$, ${\mathrm{CO}}_{2}$ and $H_{2}$O mixing ratios and precipitation rate (P). The $T_{s}$ and volumetric moisture content (θ) were measured at 2 cm depth in the mineral soil, and the relative plant available water (Rew) computed from θ using soil‐type specific field capacity and residual water contents (0.30 and 0.03 $m^{3}\;m^{‐3}$, respectively). The environmental data were gap‐filled either by regressions between other heights at the same or an adjacent tower or by regressions and look‐up tables between different variables. For consistency with climatological statistics, the annual $T_{a}$ and P were taken from adjacent (<1 km) Hyytiälä weather station operated by the Finnish Meteorological Institute. The EC and meteorological data used in this study were extracted from Mammarella et al. (2019) and Aalto et al. (2019) data releases, respectively.

#### 2.1.2 Leaf‐area index and stand growth

Trends in LAI ($m^{2}\;m^{‐2}$) were estimated using an allometric method (${\mathrm{LAI}}_{a}$) in conjunction with an optical method (${\mathrm{LAI}}_{o}$) (Suppl. S3). In the allometric method, tree inventory data were converted to needle/leaf mass per ground area using biomass equations (Marklund, 1988; Repola, 2009). Furthermore, the specific leaf areas were used to convert from leaf mass to ${\mathrm{LAI}}_{a}$ of Scots pine, Norway spruce and deciduous trees (Härkönen et al., 2015). The tree inventory data and total ${\mathrm{LAI}}_{a}$ represent the average over circular area with radius of 200 m centred on the EC tower. Tree sampling protocol is detailed elsewhere (Ilvesniemi et al., 2009). The vegetation and LAI data used in this study is based on the Kolari et al. (2022) dataset.

The ${\mathrm{LAI}}_{o}$ was determined from the ratio of below to above‐canopy PAR assuming exponential light attenuation. Only data from overcast conditions (diffuse to total PAR ratio exceeding 0.85) were used, and peak ${\mathrm{LAI}}_{o}$ values in August selected for each year. Compared with ${\mathrm{LAI}}_{a}$, ${\mathrm{LAI}}_{o}$ represents the part of the stand closest to the EC‐tower (Figure S3). Furthermore, single LiDAR‐based LAI and species stem volume rasters (measured in 2011) were used to explore how stand heterogeneity may have affected the effective LAI and species composition within the estimated annual flux footprints (Figure S4).

### 2.2 Big‐leaf framework for data and model analysis

To focus the long‐term data analysis and provide a reference for the process model scenarios, a formulation of maximum simplicity for ecosystem GPP, NEP, and ET is introduced. With LAI used as an “upscaling” kernel from leaf to canopy, Fickian diffusion representation of mass transfer on a big‐leaf GPP leads to

(1) $$\mathrm{GPP}=\mathrm{LAI}\left[c_{a}g_{s}\left(1‐\frac{c_{i}}{c_{a}}\right)\right]$$

Using a first‐order Taylor series expansion, the relative changes in GPP can be expressed and interpreted as

(2) $$\frac{\delta \text{GPP}}{\text{GPP}}\approx \underset{\text{fertilization}}{\underbrace{\left[\frac{\delta c_{a}}{c_{a}}\right]}}+\underset{\text{structural}}{\underbrace{\left[\frac{\delta \text{LAI}}{\text{LAI}}\right]}}+\underset{\text{physiological}}{\underbrace{\left[\frac{\delta g_{s}}{g_{s}}+\frac{\delta \left(1‐c_{i}/c_{a}\right)}{1‐c_{i}/c_{a}}\right]}}$$

where $g_{s}$ (mol $m^{‐2}$(leaf) $s^{‐1}$) and $c_{i}$ are the effective (canopy average) stomatal conductance per unit leaf area and inter‐cellular ${\mathrm{CO}}_{2}$ concentration, respectively. The term δLAI/LAI is later referred to as a structural adjustment, and $\delta g_{s}/g_{s}$ along with $\delta \left(1‐c_{i}/c_{a}\right)/\left(1‐c_{i}/c_{a}\right)$ as physiological adjustments reflecting acclimation and species composition, and whose impacts depend on LAI, $c_{a}$, and environmental drivers.

Likewise, ET consists of transpiration and evaporation, including contributions from wet canopy and forest floor, and is given by

(3) $$\mathrm{ET}=T_{r}+E=1.6\;\left(g_{s}\;\mathrm{LAI}\right)\;D+E$$

where D is the vapor pressure deficit and 1.6 accounts for the higher molecular diffusivity of water vapor compared with that of ${\mathrm{CO}}_{2}$. Because of the links between $T_{r}$ and GPP, the relative changes in $T_{r}$ can be decomposed into similar contributions to those of GPP as

(4) $$\frac{\delta T_{r}}{T_{r}}\approx \left[\frac{\delta \mathrm{LAI}}{\mathrm{LAI}}+\frac{\delta D}{D}+\frac{\delta g_{s}}{g_{s}}\right]=\left[\frac{\delta \mathrm{GPP}}{\mathrm{GPP}}+\frac{\delta D}{D}‐\frac{\delta c_{a}}{c_{a}}‐\frac{\delta \left(1‐c_{i}/c_{a}\right)}{1‐c_{i}/c_{a}}\right]$$

where the latter formulation arises from Equation (2). As the fertilization effect, structural and physiological adjustments, and hydroclimatic drivers (e.g., δD/D) operate on differing timescales, the underlying factors for IAV and long‐term trends may be different. Thus, whether increasing $c_{a}$ increased NEP and GPP through a direct fertilization effect, or whether hydroclimatic factors and structural shifts (e.g., LAI) are the main cause of long‐term changes in carbon and water fluxes must be addressed from this multi‐scale perspective.

#### 2.2.1 Data analysis

The mechanisms underlying trends in NEP, GPP, and ET are explored by combining statistical analysis of EC fluxes with scenarios provided by a detailed biophysical multilayer soil–vegetation–atmosphere transfer model APES (Launiainen et al., 2015, 2016). For consistency, both measured and model‐upscaled ecosystem level fluxes are analyzed in the big‐leaf framework (Knauer et al., 2018a).

The ecosystem‐level light‐use efficiency (LUE) (= GPP/ incident PAR, mmol ${\mathrm{mol}}^{‐1}$) and WUE (= GPP/ET, mmol ${\mathrm{CO}}_{2}$ ${\mathrm{mol}}^{‐1}$ $H_{2}$O) were computed as ratios of daytime (sun above horizon) sums. The water‐use characteristics were further evaluated through daytime surface conductance for ${\mathrm{CO}}_{2}$, computed as $G_{s}=ET/\left(1.6\;D\right)$ (mol $m^{‐2}\;s^{‐1}$, note ET includes also nonstomatal water fluxes), ecosystem $C_{i}/C_{a}=1‐\mathrm{GPP}/\left(G_{s}\;c_{a}\right)$ (−) and intrinsic water use efficiency $IWUE=GPP/G_{s}=c_{a}\;\left(1‐C_{i}/C_{a}\right)$ (note that capital symbols refer to big‐leaf properties). Dry‐canopy conditions were defined as periods with no rain during the past 12 h. Furthermore, carbon uptake period (CUP) start and end dates were determined according Zhu et al. (2013a). The thermal growing season was determined based on air temperature following Linderholm (2006) (Suppl. S4).

For both measured data and model scenarios, the trends in time series were evaluated using Sen's slope (Sen, 1968) and linear least‐square regression, and this was done over range of averaging periods and conditions. We adopt the term “marginally significant” for trends with *p* < .1 while *p* < .05 is used for statistical significance.

We used partial correlation coefficient ($r_{p}$) to identify drivers of IAV of fluxes and resource‐use efficiencies during different times of the year. To avoid arbitrary averaging to calendar months, we used a moving 31 day window to compute IAV and $r_{p}$. For a given day, we selected 15 previous and subsequent days and computed the arithmetic average for each year. We evaluated linear trends within the window, and define IAV as deviation from this trend. The $r_{p}$ was computed from de‐trended 31 day averages, and values with *p* < .1 recorded.

#### 2.2.2 Model scenarios

The APES model is used for analysis of the $c_{a}$ fertilization effect and structural and physiological adjustments (Equations 2 and 4) as drivers of GPP and ET trends. The goal of these model runs is to assess whether conclusions drawn from the big‐leaf representation are sensitive to non‐linearities linking biospheric fluxes to microclimatic conditions within the canopy. The model resolves the interactive effects between leaves and their micro‐climate including variable leaf area density, mean wind speed, light, and temperature variations within the canopy, among others. Full model description and test for the study site is given in Launiainen et al. (2015) (see also Figures S8 and S9).

APES computes leaf net ${\mathrm{CO}}_{2}$ exchange $A_{n}$ (μmol $m^{‐2}$(leaf) $s^{‐1}$) using Farquhar‐approach (Farquhar et al., 1980), using the specific formulation of the Farquhar‐model and temperature adjustments to its kinetic rate constants from Medlyn et al. (2002) temperature. The co‐limitation of Rubisco‐ and RuBP‐regeneration limitations are accounted for using standard formulations Collatz et al. (1990) relationship. The leaf‐scale stomatal conductance (mol$g_{s}$ $m^{‐2}$ (leaf) $s^{‐1}$) for ${\mathrm{CO}}_{2}$ is related to photosynthetic ${\mathrm{CO}}_{2}$ demand assuming plants maximize carbon assimilation for a given amount of water loss per unit leaf area under RuBP regeneration limitations (Medlyn et al., 2012)

(5) $$g_{s}=g_{o}+\left[1+\frac{g_{1}\left(\theta \right)}{\sqrt{D_{l}}}\right]\frac{A_{n}}{c_{s}}$$

where $c_{s}$ is the ${\mathrm{CO}}_{2}$ mixing ratio at leaf surface, $g_{o}$ (mol $m^{‐2}$ (leaf) $s^{‐1}$) is residual conductance and $g_{1}$ (${\mathrm{kPa}}^{0.5}$) a parameter proportional to the marginal WUE reflecting plant water use strategies (Lin et al., 2015; Medlyn et al., 2012). The leaf‐scale $A_{n}$ and $g_{s}$ are solved separately for sunlit and shaded leaves of each vascular plant type (here pine, spruce, deciduous, and understory shrubs) exposed to different radiation regimes at each canopy layer (here 100 layers). The solution is coupled with the leaf energy balance approximating leaves as flat plates exposed to parallel free and forced convection to compute leaf boundary layer conductance (Campbell & Norman, 1998; Launiainen et al., 2015). For each plant type, the maximum carboxylation capacity at a reference leaf temperature set to $25^{\circ }$C ($V_{cmax25}$) is assumed to vary with seasonal cycle of photosynthetic capacity, leaf nitrogen ($N_{l}$) and soil water availability. For the latter, $V_{cmax25}$ and $g_{1}$ are non‐linear functions of plant available water (Keenan et al., 2010; Zhou et al., 2013) parameterized according to shoot gas‐exchange data from the study site (Launiainen et al., 2015). The maximum electron transport rate ($J_{max25}$) and dark respiration rate at $25^{\circ }$C ($r_{d25}$) are described proportional to $V_{cmax25}$.

Microclimatic modules of APES solve short‐ and long‐wave radiation, interception of rainfall and evaporation from wet leaves, and the vertical variations of mean wind speed (U) and scalar quantities ($T_{a}$, $c_{a}$, $H_{2}$O) iteratively with leaf gas exchange. The forest floor is assumed to be covered by moss with a prescribed thickness. The model is forced by 1/2 h meteorological variables measured above the canopy, and measured $T_{s}$ and θ are used as lower boundary conditions for the canopy water and heat balance. The bulk soil respiration, including heterotrophic and autotrophic components, provides the lower boundary condition for the canopy ${\mathrm{CO}}_{2}$ budget (Launiainen et al., 2015).

Model scenarios were performed using measured meteorological time series 2001–2017 along with time series of $c_{a}$ and LAI sequentially added. In addition, the sensitivity to $N_{l}$ was tested assuming $V_{cmax25}$ scales with $N_{l}$ (Kattge et al., 2009) (Suppl. S7). The model scenarios allow assessing the effects of single drivers, as well as the interactive effects between $\delta c_{a}/c_{a}$, δLAI/LAI, and the physiological adjustments $\delta g_{s}/g_{s}$, and $\delta \left(1‐c_{i}/c_{a}\right)/\left(1‐c_{i}/c_{a}\right)$ (Equations 2 and 4). In addition, the model is able to discern between stomatal and non‐stomatal water sources as components of ET. Except variables altered in scenarios, parametrization (Tables S2 and S3) follows Launiainen et al. (2015) and Leppä et al. (2020).

## 3 RESULTS

To address connections between the 40 ppm increase in $c_{a}$ from 2001 to 2017 and the main drivers leading to enhancements in NEP and GPP, data analyses and temporal variability in external drivers are first reported at seasonal and inter‐annual time scales (i.e., timescales commensurate with changes in LAI and physiological traits). Averaging over seasonal and annual timescales tend to ameliorate the large hydroclimatic variability affecting stomatal conductance and net assimilation rates, which enables robust statements about the relative contribution of the direct $c_{a}$ fertilization effect versus structural or physiological adjustments. To further constrain these structural and physiological findings, the drivers and concomitant changes in ET and resource use efficiencies are also presented.

### 3.1 Seasonal and inter‐annual variability

**FIGURE 1:**
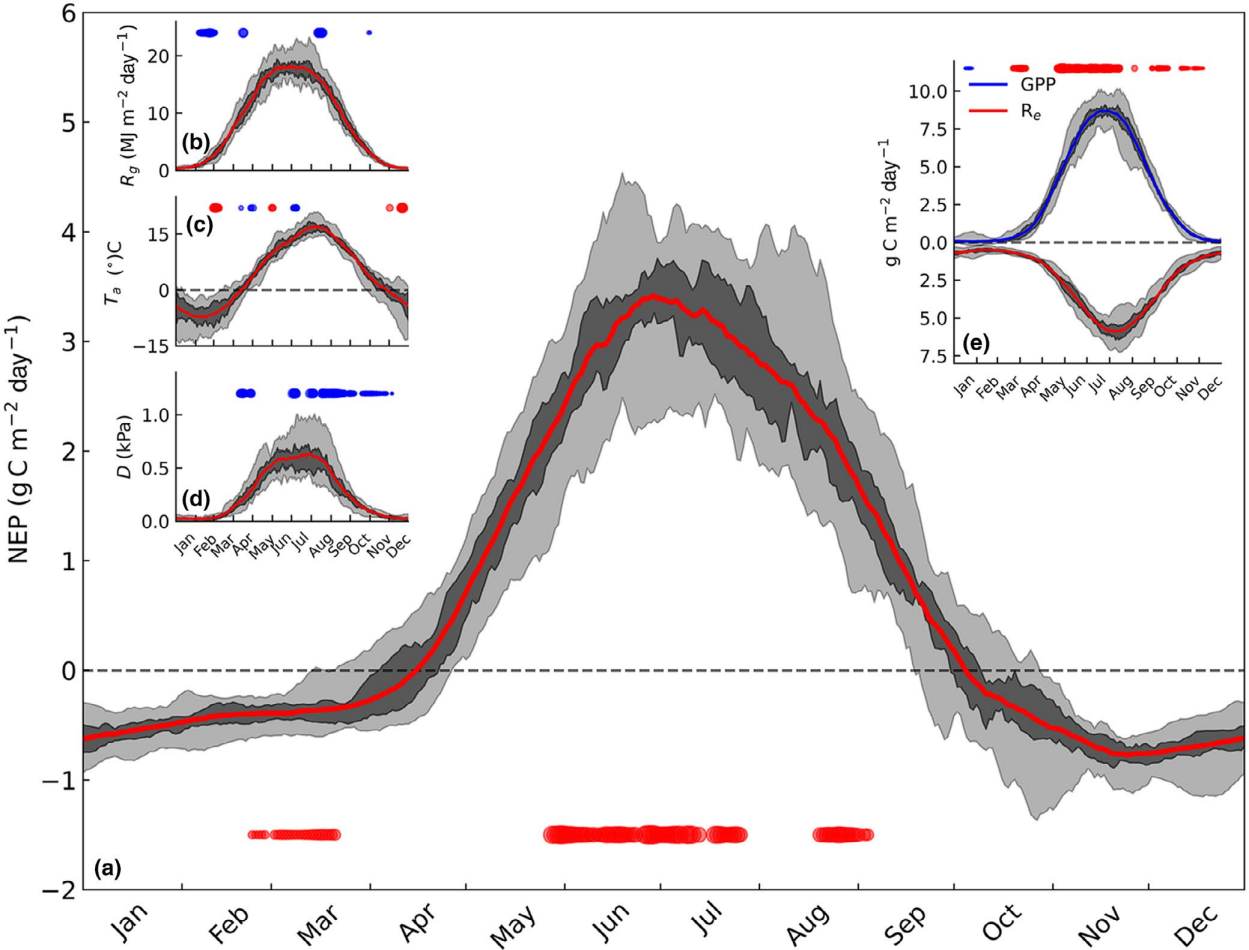
Seasonal patterns of net ecosystem productivity (NEP), gross primary productivity (GPP), and ecosystem respiration ($R_{e}$) and environmental conditions in 2001–2017. The lines show 31‐day moving average, dark shades are 25/75th percentiles, and light shade are the entire variability range. In each panel, positive/negative trends (*p* < .1) are shown by red/blue circles, respectively. The size is relative to maximum absolute trend value. The $R_{e}$ trends were all nonsignificant

To illustrate the seasonal cycle, the ensemble variations in NEP and its two component fluxes (GPP and $R_{e}$) are shown in Figure 1, where ensemble averaging is conducted over all years at a given day. The GPP and $R_{e}$ exhibit expected seasonal patterns but with notable phase shifts. The GPP peaks from late June to mid‐August, and is skewed to the right compared with incoming global radiation (Figure 1a and b). This is a combined effect of dormancy recovery, seasonal course of LAI and approximately one month timelag between peaks of $R_{g}$ and $T_{a}$ (Figure 1b and c). As the annual pattern of $R_{e}$ follows closely that of $T_{s}$ (not shown), the asymmetric cycles of the component fluxes lead to NEP peak from late May to early July, while highest net ${\mathrm{CO}}_{2}$ emissions occur in late autumn (Figure 1a and e).

**FIGURE 2:**
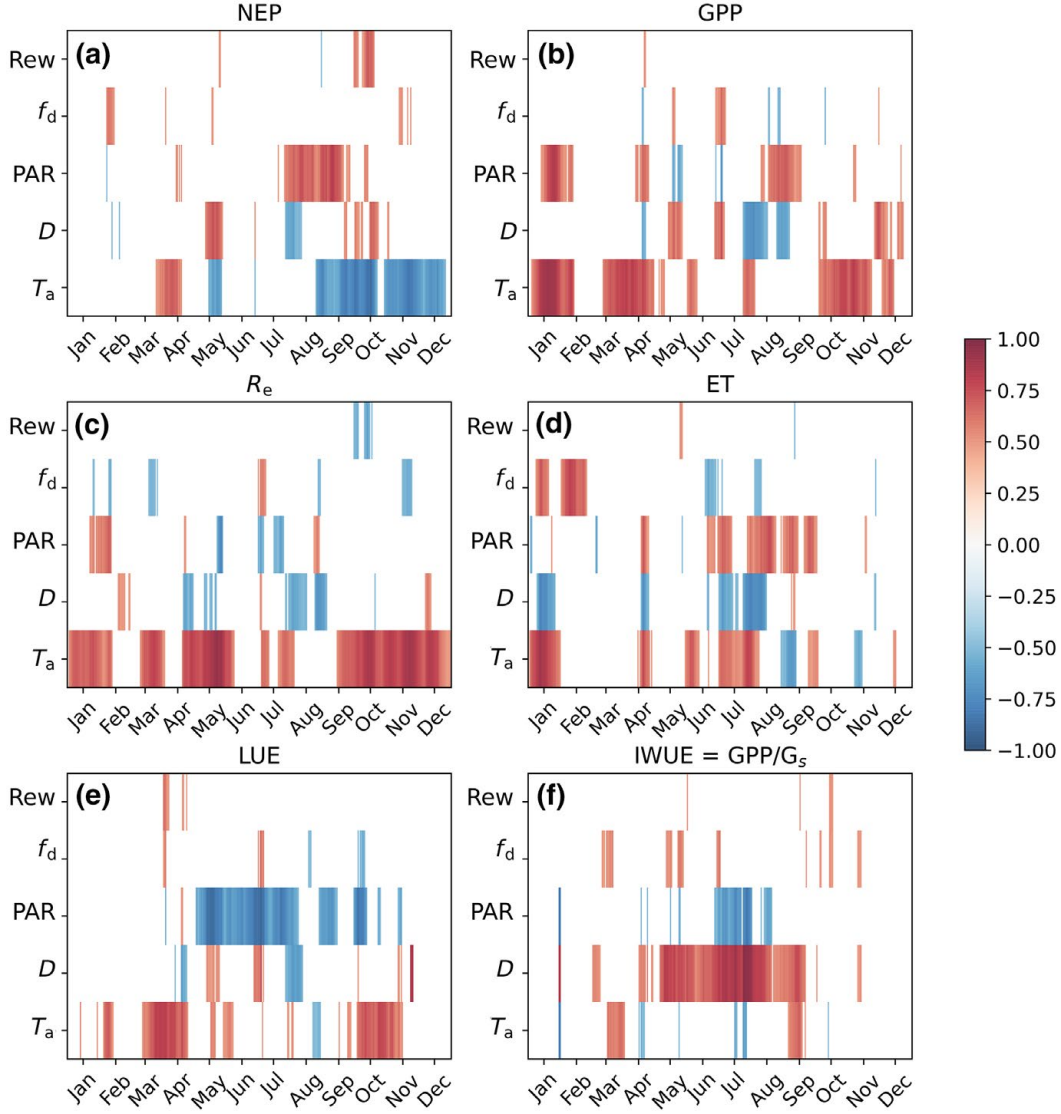
Partial correlation coefficients ($r_{p}$) between net ecosystem productivity (NEP) (a), gross primary productivity (GPP) (b), $R_{e}$ (c), evapotranspiration (ET) (d), light‐use efficiency (LUE, e) and intrinsic water‐use efficiency (IWUE, f), and their potential environmental drivers. $T_{a}$ air temperature, D vapor pressure deficit, PAR photosynthetically active radiation, $f_{d}$ its diffuse fraction, Rew plant available water. The colors show marginally significant correlations (*p* < .1, corresponding to $|r_{p}|>0.41$ for 17‐year timeseries) in monthly window

Partial correlation analysis was applied to monthly moving windows so as to link the de‐trended IAV of carbon and water exchange rates to variations in the environmental drivers (Figure 2). The de‐trending is applied here to remove potential effects of structural changes (δLAI), trait plasticity and direct effect of $c_{a}$ to focus on the IAV caused by physiological adjustments to meteorological drivers (Equation 2). Strong negative correlation between NEP and $T_{a}$ (Figure 2a) was observed in September–December, as there is stronger positive relation between $R_{e}$ and $T_{a}$ (Figure 2c) at low light levels— at least when compared with GPP (Figure 2b). In March–mid‐April, GPP and LUE correlate well with $T_{a}$ (Figure 2b and e) compared with$R_{e}$, leading to positive relation between NEP and temperature. In this period, the forest floor is typically snow covered and soil respiration does not react to $T_{a}$ variations. The situation is reversed in early May, when a strong positive relation between IAV of $R_{e}$ and $T_{a}$ explains the negative correlation between NEP and temperature (Figure 2a–c).

In Jun–July, IAV of NEP was not correlated with any of the environmental variables studied. In late July–early August, NEP, GPP, and LUE decreased with increasing D, indicative of stomatal limitations (Figure 2a,b,e). In May, when plant available water (Rew) was ample for all years (not shown), these correlations were positive. In late summer–early autumn, NEP and GPP had a strong positive correlation with PAR (Figure 2a and b). As expected, PAR was also the main driver of LUE variations and LUE decreased with increasing PAR throughout the growing season (Figure 2e).

With the exception of late Aug–early Sep, the IAV of ET correlated positively with $T_{a}$ as ET is driven by atmospheric evaporative demand. Compared with GPP, the positive relation with PAR and negative correlation with its diffuse fraction and D are more frequent (Figure 2d). The intrinsic WUE (GPP/$G_{s}$) increased with D throughout the summer and was negatively correlated with PAR, indicative of stronger sensitivity of $G_{s}$ than GPP to IAV of D and light availability (Figure 2f). Neither carbon nor water fluxes correlated with relative plant available water. This finding may be explained by the fact that during the 17 year record, soil water content decreased below a threshold markedly affecting shoot gas‐exchange (physiological drought) only for a restricted period in late summer 2006 (not shown).

### 3.2 Annual and seasonal trends and balances

#### 3.2.1 Climatic conditions

**FIGURE 3:**
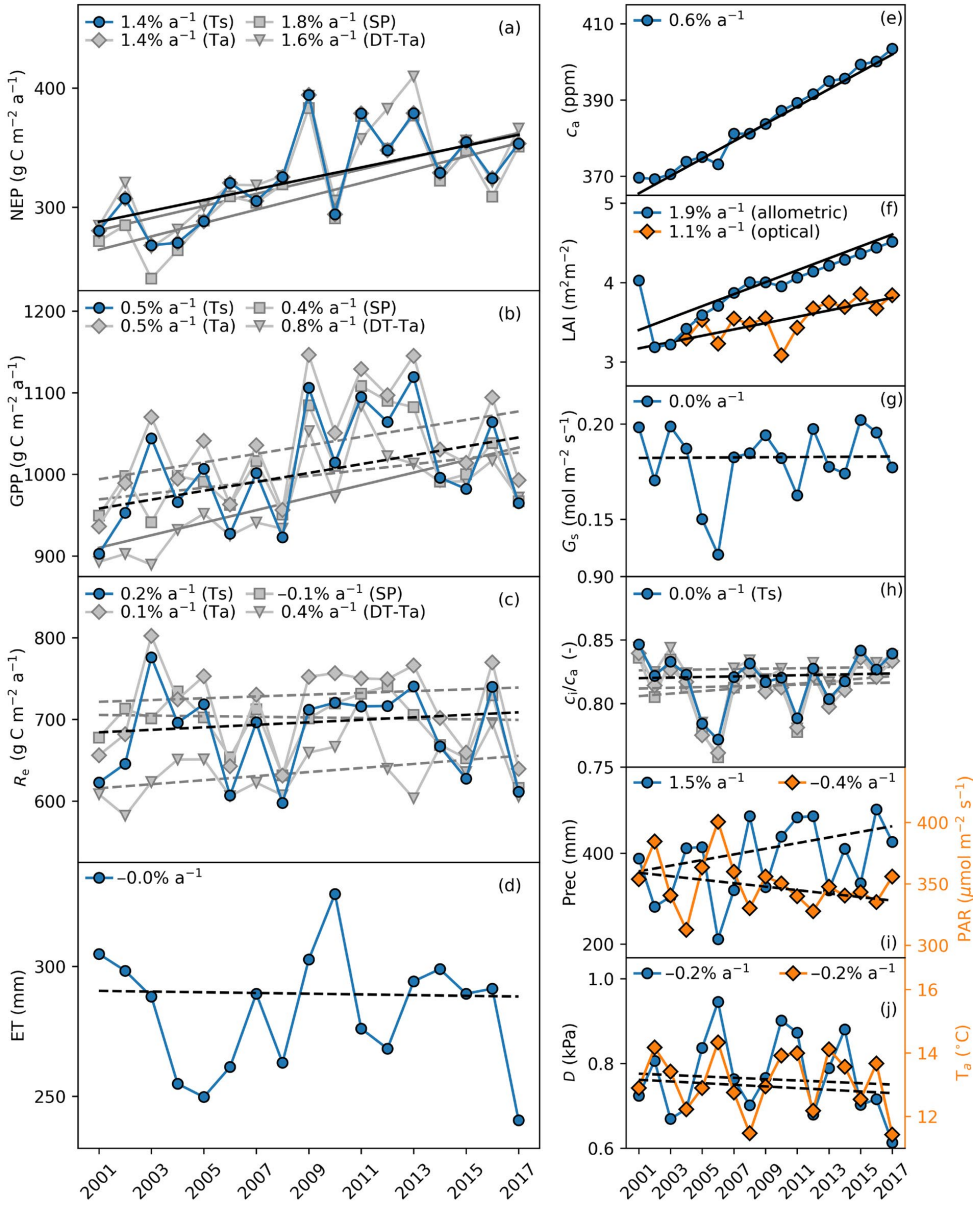
Net ecosystem productivity (NEP, a), gross primary productivity (GPP, b), ecosystem respiration ($R_{e}$, c) and evapotranspiration (ET, d) for May‐Sept period in 2001–2017 (left panels). The right panels show mean ambient ${\mathrm{CO}}_{2}$ mixing ratio ($c_{a}$, e), leaf‐area index (LAI, f), photosynthetically active radiation (PAR, i), air temperature ($T_{a}$, j), accumulated precipitation (Prec, i), and mean daytime (sun above horizon) vapor pressure deficit (D, j). The surface conductance ($G_{s}$, g) and ratio of internal to ambient ${\mathrm{CO}}_{2}$ ($c_{i}$/$c_{a}$, h) depict daytime medians. Continuous black line denotes statistically significant trend (Sen's slope, *p* < .05). NEP, GPP and $R_{e}$ based on four different partitioning methods are shown. For trend values, see Table 2

The marked IAV of meteorological conditions drives the IAV of carbon and water exchanges. However, apart of $c_{a}$ (+0.6% $a^{‐1}$), only a few weak trends in environmental conditions were found over the 17‐year period studied (Figure 1b–d; Figure 3e–j; Tables 1 and 2). A weak and statistically non‐significant increase in May–Sept precipitation and decrease in solar radiation and D were observed (Figure 3i; Tables 1 and 2). There was a marginal shift towards more humid and cloudier autumns and mid‐winter as time progressed. A marginally significant advancement in start date of net carbon uptake (−1.0 d $a^{‐1}$), occurring on average April 17th, was found. However, no change in end of CUP or timing of the thermal growing season was observed (Table 2; Figure S5).

**TABLE 2:** Annual and warm season (May–September) trends

	mean (±SD)	Trend (Sen, $a^{‐1}$)	Trend (lin., $a^{‐1}$)	$r^{2}$	*p*
Annual					
${\mathrm{NEE}}_{\mathrm{Ts}}$ (g $m^{‐2}$)	252 (42)	6.4	6.7	.66	.000
${\mathrm{NEE}}_{\mathrm{SP}}$ (g $m^{‐2}$)	239 (48)	7.0	7.7	.66	.000
${\mathrm{GPP}}_{\mathrm{Ts}}$ (g $m^{‐2}$)	1148 (71)	8.0	8.2	.34	.014
${\mathrm{GPP}}_{\mathrm{Ta}}$ (g $m^{‐2}$)	1180 (70)	8.2	8.0	.33	.015
${\mathrm{GPP}}_{\mathrm{SP}}$ (g $m^{‐2}$)	1148 (61)	7.3	6.6	.31	.021
${\mathrm{GPP}}_{\mathrm{DT}}$ (g $m^{‐2}$)	1117 (67)	10.1	10.1	.58	.000
$R_{e,Ts}$ (g $m^{‐2}$)	896 (53)	1.3	1.5	.02	.599
$R_{e,Ta}$ (g $m^{‐2}$)	928 (52)	1.5	1.3	.02	.621
$R_{e,SP}$ (g $m^{‐2}$)	909 (35)	−0.6	−1.0	.02	.567
$R_{e,DT}$ (g $m^{‐2}$)	809 (41)	2.9	3.5	.18	.091
ET (mm)	348 (26)	1.8	1.8	.12	.170
$T_{a}$ (°C)	4.4 (0.8)	0.07	0.04	.06	.345
$T_{s}$ (°C)	5.7 (0.4)	0.01	0.00	.00	.900
D (kPa)	0.26 (0.04)	−0.003	−0.004	.28	.030
PAR (μ mol $m^{‐2}\;s^{‐1}$)	200.8 (11.2)	−1.1	−1.1	.22	.055
$c_{a}$ (ppm)	390.4 (11.8)	2.3	2.3	.98	.000
GS start (doy)	123 (12)	0.71	0.97	.12	.10
GS end (doy)	294 (11)	−0.03	0.3	.02	.63
GS length (days)	172 (15)	−0.9	−0.7	.05	.38
SCU (doy)	98 (12)	−0.87	−1.17	.22	.055
ECU (doy)	266 (13)	0.53	0.48	.03	.490
CUP (days)	169 (20)	1.61	1.64	.17	.096
NEP gaps (%)	33.6 (5.6)	0.007	0.007	.004	.80
EBC (−)	0.85 (0.04)	0.006	0.005	.40	.011
May–September					
${\mathrm{NEE}}_{\mathrm{Ts}}$ (g $m^{‐2}$)	325 (39)	4.56	5.10	.44	.004
${\mathrm{NEE}}_{\mathrm{SP}}$ (g $m^{‐2}$)	317 (42)	5.60	5.71	.47	.002
${\mathrm{GPP}}_{\mathrm{Ts}}$ (g $m^{‐2}$)	1008 (66)	5.45	5.37	.17	.101
${\mathrm{GPP}}_{\mathrm{Ta}}$ (g $m^{‐2}$)	1040 (66)	5.20	5.22	.16	.110
${\mathrm{GPP}}_{\mathrm{SP}}$ (g $m^{‐2}$)	1011 (53)	3.59	4.25	.16	.106
${\mathrm{GPP}}_{\mathrm{DT}}$ (g $m^{‐2}$)	970 (56)	7.67	7.84	.50	.002
$R_{e,Ts}$ (g $m^{‐2}$)	683 (55)	1.53	0.27	.00	.924
$R_{e,Ta}$ (g $m^{‐2}$)	716 (54)	1.09	0.12	.00	.965
$R_{e,SP}$ (g $m^{‐2}$)	694 (37)	−0.38	−1.46	.04	.447
$R_{e,DT}$ (g $m^{‐2}$)	638 (37)	2.48	2.69	.13	.150
ET (mm)	282 (23)	−0.14	−0.23	.00	.845
LUE (mmol ${\mathrm{mol}}^{‐1}$)	17.83 (1.63)	0.16	0.16	.23	.049
WUE (mmol ${\mathrm{mol}}^{‐1}$)	5.34 (0.48)	0.05	0.04	.15	.123
$T_{a}$ (°C)	13.1 (0.9)	−0.03	−0.03	.02	.587
$T_{s}$ (°C)	10.4 (0.6)	0.04	0.03	.05	.382
D (kPa)	0.77 (0.09)	0.00	0.00	.02	.631
PAR (μ mol $m^{‐2}\;s^{‐1}$)	349.5 (20.8)	−1.42	−1.16	.08	.273
P (mm)	383 (83)	6.22	6.55	.16	.111
$G_{s}$ (mol $m^{‐2}\;s^{‐1}$)	0.18 (0.02)	0.00	0.00	.02	.568

#### 3.2.2 Leaf‐area index and flux footprint

The stand was partially thinned in January–February 2002, resulting to ca. 20% drop in LAI. According to an earlier study (Vesala et al., 2005), thinning did not affect ecosystem carbon and water exchange, which they attributed as compensatory carbon uptake and ET from the understory. Since 2002, LAI consistently increased but the estimated growth rate varies depending on the LAI estimation method (Figure 3f). The increase of stand height and concomitant roughness length lead to decrease in mean wind speed and non‐significant increase in friction velocity at 23 m above the forest floor (Figure S2). According to the analytical footprint model (Kljun et al., 2015), this led to the 80% footprint boundary shifting progressively closer to the EC tower as expected (Figure S3), and the footprint area shrank by ca. 70% (Figure S4). We used a snapshot of spatial LAI and species composition maps from 2011 and showed that the footprint trend has likely lead to increasing contribution of Scots pine on the observed fluxes toward the end of the 17‐year period (Figure S4). The footprint change affects also LAI estimates for the ecosystem observed from the EC tower. Due to the initial stand heterogeneity, shrinking footprint would decrease the footprint‐averaged LAI by ca. 10% during the period if LAI development due to forest growth is neglected (Figure S4). This effect is, however, much smaller than observed LAI growth rate but suggests that ${\mathrm{LAI}}_{a}$ trend that assumes time‐constant footprint may overestimate the LAI development within the dynamic EC footprint. To account for the LAI trend uncertainty, both ${\mathrm{LAI}}_{o}$ and ${\mathrm{LAI}}_{a}$ trends were included in model scenarios.

#### 3.2.3 Trends in carbon and water exchange

The site was a persistent annual carbon sink with NEP ranging from 152 to 309 g C $m^{‐2}$ (mean 252 g C $m^{‐2}$ $a^{‐1}$; Tables 1 and 2). The annual mean GPP was 1117–1180 and $R_{e}$ was 809–928 g C $m^{‐2}$ $a^{‐1}$ depending on the flux‐partitioning method. During the 2001–2017 period, annual NEP increased at a mean rate of 6.4–7.0 g C $m^{‐2}$ $a^{‐1}$ (or +2.5 % $a^{‐1}$ of long‐term mean, *p* < .01). This increase was due to a positive trend in GPP, whose magnitude varied from +8.0 to 10.0 g C $m^{‐2}$ $a^{‐1}$ (+0.6 to +0.9% $a^{‐1}$, *p* < .05) depending on the flux partitioning method. The trend in annual $R_{e}$ was nonsignificant, from −0.6 to +2.9 g C $m^{‐2}$ $a^{‐1}$ (Table 2). The IAV of NEP and GPP, estimated as the standard deviation of the linearly de‐trended timeseries, were 26 and 50 g C $m^{‐2}$ $a^{‐1}$ (mean over partitioning methods), respectively.

**FIGURE 4:**
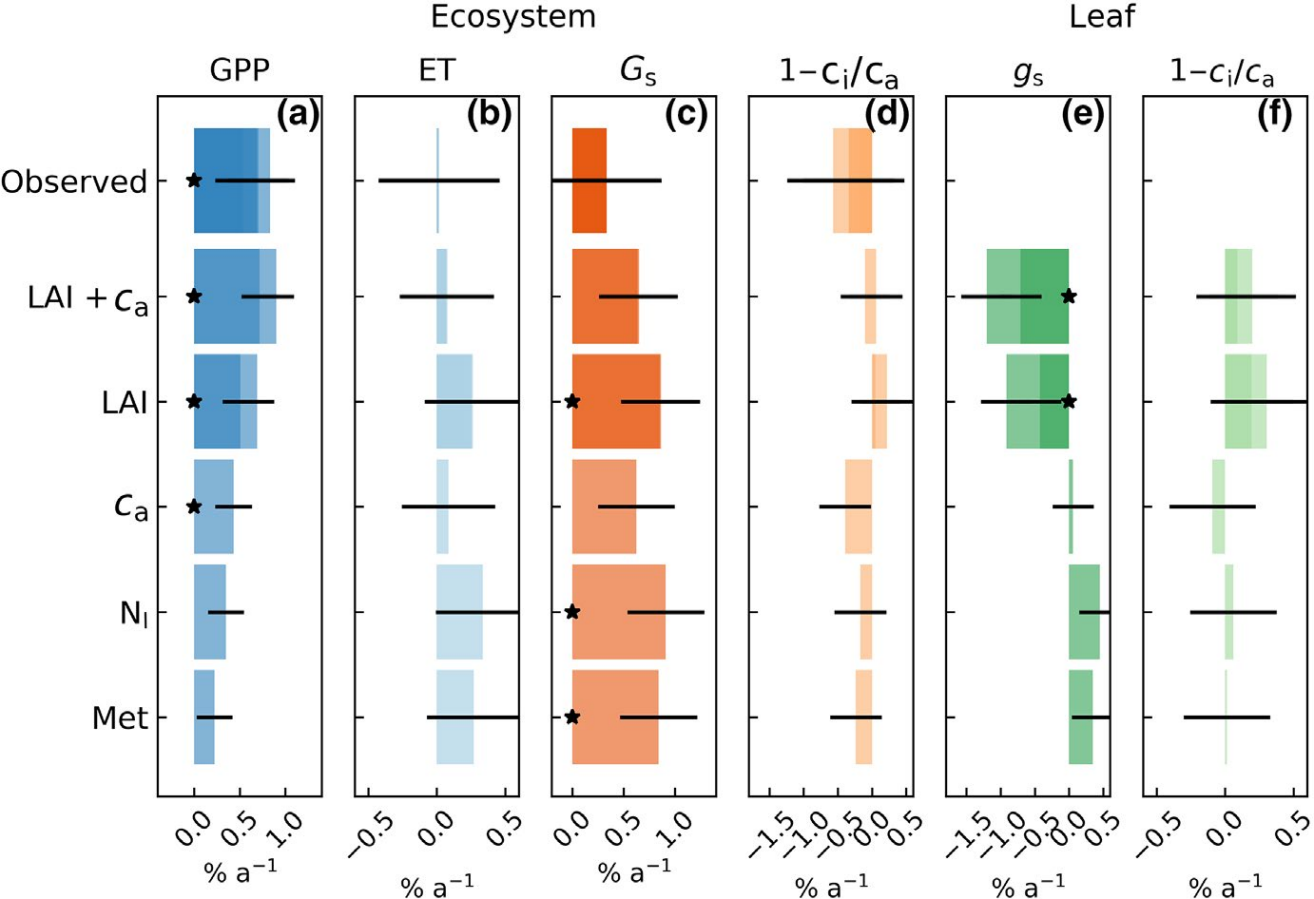
Observed and modelled trends in gross primary productivity (GPP, a), evapotranspiration (ET, b), ecosystem surface conductance ($G_{s}$, c) and $C_{i}/C_{a}$ (d), and modelled trends in canopy mean stomatal conductance ($g_{s}$, e) and leaf $c_{i}/c_{a}$ (f). The trends are slopes (±SE) of linear regression to yearly sums/averages of daytime dry‐canopy conditions in May–September. Trends are shown as % of the 2001–2017 mean, and (*) denotes statistically significant (*p* < .05) slope. The relative trends in $\delta c_{a}/c_{a}$ = 0.6% $a^{‐1}$ and δ LAI/LAI = 1.1–1.9% $a^{‐1}$ (Figure 3). The shades correspond to different flux‐partitioning and LAI‐trend estimates

The positive trend in annual NEP was attributed mainly to increasing growing‐season GPP. Strongest trends occurred in the first half of the growing season (May–July, Figure 1a and e). The increase of the warm season (May–Sept) cumulative NEP (+4.6 to +5.6 $m^{‐2}$ $a^{‐1}$) and GPP (+3.6 to +7.7 g C $m^{‐2}$ $a^{‐1}$ m; Figure 3a–c and Tables 1 and 2) contributed ca. 3/4 and 2/3 on the annual increase, respectively. The cumulative GPP and NEP increased significantly also in the cool season (October–April) (+2.4 to 2.6 g C $m^{‐2}$ $a^{‐1}$, *p* < .05; Tables 1 and 2), mostly as a response to rising wintertime air temperature (Figure 2a–c). The IAV of ET, surface conductance, ecosystem $C_{i}/C_{a}$ and WUE was notable but these variables showed no temporal trends (Figure 3d,g,h and Figure 4b–d; Tables 1 and 2).

We further considered 1st of July to 10th of August as a period when ecosystem properties at the site tend to be stationary in terms of LAI, phenology and edaphic processes. Only data measured in dry‐canopy conditions under sufficiently moist soils were included to minimize the non‐stomatal water sources and to exclude possible carry‐over effects of soil moisture limitations, respectively. To standardize for environmental conditions, data were further clustered into PAR classes, trends evaluated separately for each class and ecosystem light‐response curve fitted to the binned averages (Suppl. S5). A marginally significant increase of ecosystem GPP (+1.0% $a^{‐1}$), LUE (+0.4–0.6% $a^{‐1}$) and WUE (+0.9–1.3% $a^{‐1}$) at PAR > 700 μmol $m^{‐2}$ $s^{‐1}$ was observed (Figure S7). These trends represent increase of ecosystem carbon uptake capacity in the most favorable conditions and are, in relative sense, stronger than the growing‐season average trends. However, no change in ET, $G_{s}$ or ecosystem $C_{i}/C_{a}$ were found in any of the light classes analyzed (Figures S6 and S7).

### 3.3 Contribution of climatic and structural changes to observed gas exchange trends

**FIGURE 5:**
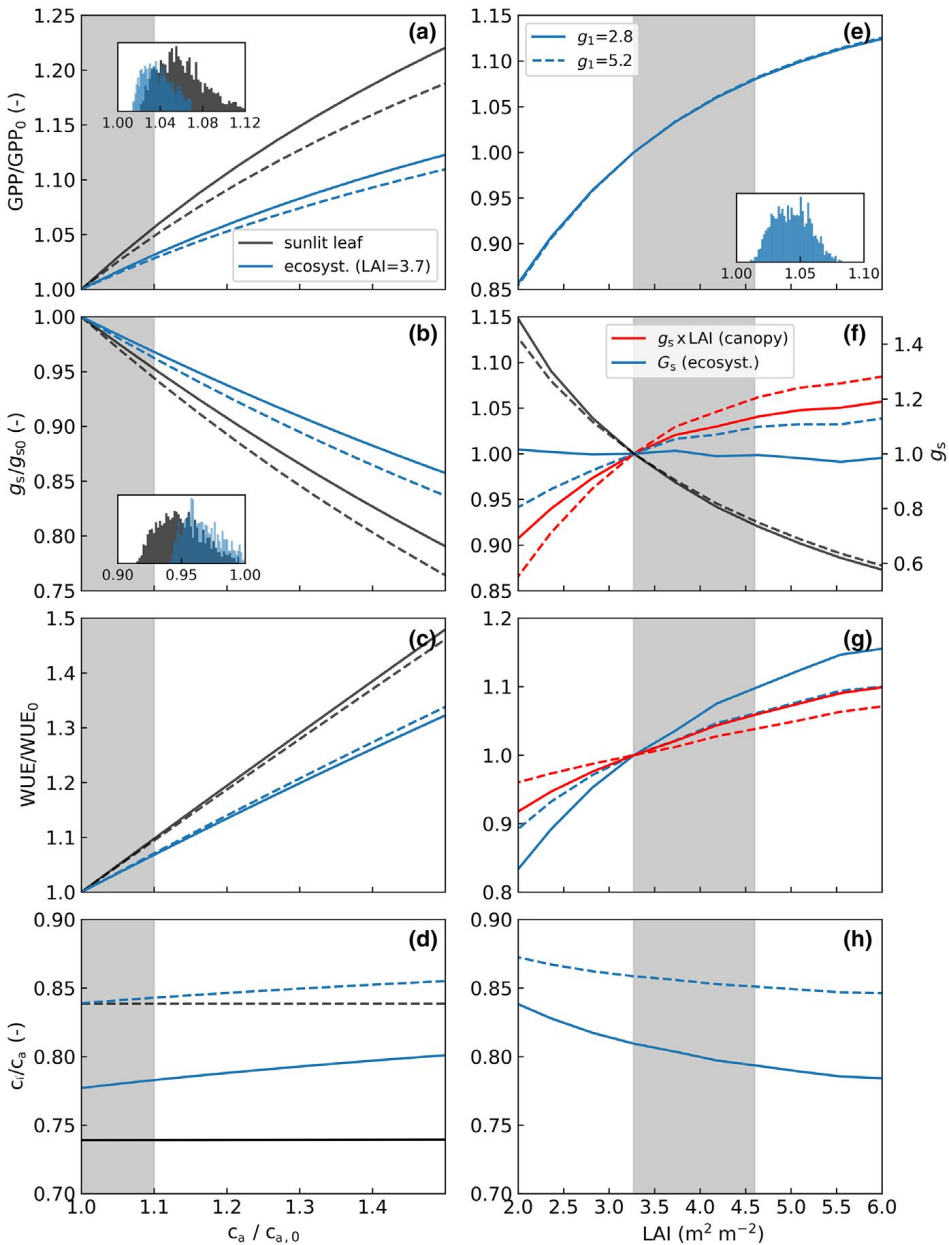
Modeled leaf and ecosystem response of gross primary productivity (GPP), stomatal/surface conductance ($g_{s}$), water‐use efficiency (WUE), and $c_{i}/c_{a}$ to atmospheric ${\mathrm{CO}}_{2}$ ($c_{a}$) (left). The right panels show ecosystem scale response to lead‐are index (LAI) (right). The values are means of daytime dry‐canopy values (PAR > 100 μmol $m^{‐2}$ $s^{‐1}$, no rain in previous 24 h) over a typical growing season (2008). The leaf values represent a sunlit leaf at top of the canopy, and the three first rows are normalized by response at $C_{a,0}$ = 375 ppm (left) or LAI (right) observed at the study site, whereas the gray shaded area shows the respective ranges. The solid/dashed lines represent water use strategies ($g_{1}$) characteristic for coniferous and deciduous trees, respectively. In (f) $g_{s}$ is canopy average stomatal conductance, $g_{s}\times LAI$ represents canopy conductance and $G_{s}$ is the ecosystem surface conductance. The insets show pdfs of instantaneous fertilization and LAI effects arising from microclimatic variability during the growing season

The EC data suggest May–September GPP increased roughly proportionally to$c_{a}$; that is$\delta GPP/GPP\;\approx \;\delta c_{a}/c_{a}$. However, ecosystem ET and surface conductance did not decrease (Figure 3d and g; Tables 1 and 2) as would be expected for a direct fertilization effect. To interpret such behavior, we used the APES model to distinguish between direct $c_{a}$ effect and structural adjustments and to identify any physiological adjustments (Equations 2 and 4) causing the long‐term trends. We show the model scenarios specific for the study site in Figure 4 and generalize the results to broader ranges of $c_{a}$ and LAI in the absence of inter‐annual meteorological variability in Figure 5.

We first held $c_{a}$ as constant, and set LAI and $N_{l}$ to their mean values to analyze the effect of meteorological drivers (Met) during 2001–2017. The results indicated environmental factors are responsible for much of the IAV (Figures 2 and 3). They did, however, not significantly contribute to long‐term trends, with the exception of increase of $G_{s}$ (Figure 4g) as a response to a decreasing D and increasing precipitation (Figure 3i and j). Compared to this baseline scenario, including $c_{a}$ fertilization lead to an increase of GPP, while $G_{s}$ and $C_{i}/C_{a}$ and their effective leaf‐level counterparts ($g_{s}$ and $c_{i}/c_{a}$) were reduced as expected (Figure 4). Accounting for the structural adjustments (δLAI/LAI, +1.1–1.9% $a^{‐1}$) lead to positive GPP trend closer to the observed value and had negligible impact on ET or $G_{s}$ compared with the baseline scenario. However, neither mechanism alone could explain the concomitant increase of GPP and the invariant ET and $G_{s}$.

When both LAI changes and $c_{a}$ increase were accounted for, the model predictions agreed reasonably with the observed trends (or their lack of, Figure 4a–d). The measured δGPP/GPP and δLAI/LAI are both uncertain (Figure 3), but even accounting for this the LAI development appears as stronger driver and can explain 55–70% of the observed GPP trend (Figures 4a and 5a,e).

We further tested how increasing leaf $V_{\mathrm{cmax}}$ would affect ecosystem‐level fluxes. Data from the ICP Level 2 forest condition monitoring plots in Finland show consistent increase of Scots pine $N_{l}$ (+0.23 mg $g^{‐1}$ $a^{‐1}$, or ca 1.8% $a^{‐1}$, *p* < .01, Figure S11) during 1995–2013 period Merila and Jortikka (2017). The reason for such increase is not entirely understood as bulk nitrogen deposition has not significantly changed (Figure S12). Assuming $V_{cmax25}$ scales with $N_{l}$ as reported for boreal coniferous forests (Kattge et al., 2009), the resulting GPP trend from $N_{l}$ would be roughly 2/3 of the direct $c_{a}$ effect (Figure 4a). Moreover, increasing leaf photosynthetic capacity is predicted to increase leaf and ecosystem water fluxes counter to what was observed (Figure 4b,c,e).

## 4 DISCUSSION

### 4.1 **Response to increasing** $c_{a}$

The model predictions show the expected stimulation of photosynthesis, decreasing stomatal/surface conductance and near‐constant leaf $c_{i}/c_{a}$ and ecosystem $C_{i}/C_{a}$ as $c_{a}$ rises (Figures 4 and 5). These result in well‐established enhancement of WUE at both leaf and ecosystem scale (De Kauwe et al., 2013; Katul et al., 2009; Medlyn et al., 2001). The results, however, suggest the direct $c_{a}$ effect at ecosystem scale can be roughly a factor of two weaker compared to a well‐illuminated leaf in the upper canopy (Figure 5a–c). This is similar to what was recently found for EucFACE (Yang et al., 2020) and can be primarily attributed to increasing RuBP‐regeneration limitations when moving from sunlit leaf to ecosystem scale (Ewert, 2004; Yang et al., 2020). We note the degree of downregulation of $V_{\mathrm{cmax}}$ and varying $V_{\mathrm{cmax}}$ to $J_{\max}$ ratio through acclimation to canopy light gradient and increasing $c_{a}$ are likely to affect ecosystem response to elevated $c_{a}$ (Yang et al., 2020) and should be further explored. Moreover, alternative formulations of stomatal optimality and varying water‐use strategies (Figure 5a and b) can yield different fertilization effects (Katul et al., 2009; Medlyn et al., 2012). Addressing these was out of the scope of this work.

In the simulations, we did not account for down‐regulation of photosynthetic capacity with $c_{a}$ (Long et al., 2004) and ignored feedbacks from δLAI to soil water content (via plant water use and rainfall interception) by using observed soil water content as model boundary condition. These simplifications are considered feasible for the $\delta c_{a}$ during the study period, and given the rare occurrence of soil moisture limitations (once in the 17 years studied) at the site.

The growing‐season integrated sensitivity of GPP to $c_{a}$ is comparable to recent predictions for boreal coniferous forests by CABLE land‐surface model, showing δGPP ~ 15% for 1.6$\times \;c_{a}$, (Haverd et al., 2020). The weaker $c_{a}$ effect in boreal than in temperate forests can presumably be attributed to the relatively low‐fertility (i.e.$V_{\mathrm{cmax}}$) and relatively low temperatures during the growing season (Hickler et al., 2008 [CO_2_]; He et al., 2017). The microclimatic variability results to range of instantaneous effects to elevated $c_{a}$ both at leaf and ecosystem scales, depicted as pdfs in Figure 5a,b,e. These pdfs underlie the expected long‐term (e.g., growing season) response. The results emphasize that comparing or interpreting $c_{a}$ effects across studies and biomes must pay increasing attention to both spatial (leaf to ecosystem) and temporal (seasonal to inter‐annual) upscaling.

### 4.2 Response to leaf‐area index

The non‐linear increase of GPP with forest LAI is a common finding in chrono‐sequence studies (Amiro et al., 2010; Goulden et al., 2011), multi‐site syntheses (Lagergren et al., 2008; Launiainen et al., 2016; Lindroth et al., 2008) and modeling studies (Wu et al., 2013; Launiainen et al., 2016). For the observed ${\mathrm{LAI}}_{a}$ range from ca. 3.2 to 4.5 $m^{2}\;m^{2}$, the model predictions suggest ca. 7% increase in GPP while maintaining a near‐constant $G_{s}$ and ET (Figure 5e and f). The decrease of canopy mean leaf $g_{s}$ with LAI (physiological adjustment due light limitations, Equations 2 and 4) means the canopy conductance ($g_{s}\times LAI$) remains more conservative to LAI changes (Roberts, 1983) than that of GPP, leading to the increased transpiration use efficiency of denser forests (Figure 5g). Furthermore, any increase in canopy conductance with LAI tends to be compensated by reduced evaporation from the forest floor (energy limitations) resulting in a surprisingly stable dry‐canopy $G_{s}$ across wide range of forest LAIs (Figure 5f and g) (Launiainen et al., 2016).

In support of this, the long‐term observations showed marginally significant increases in ecosystem WUE under ample light during the peak GPP period (Figure S7). However, no trend in WUE was detected over the whole growing season. This can be attributed to (i) weaker sensitivity of GPP and $G_{s}$ to $c_{a}$ and LAI variations in less favorable environmental conditions (see pdfs in Figure 5a,b,e) and (ii) strong IAV in D (Figure 3j; Table 1) that likely masks subtle trends in plant response to $c_{a}$ or LAI. The modeled interception evaporation increased with LAI as reported earlier (Leppä et al., 2020; Pitman, 1989) and dominated IAV of ET (not shown). Due to high uncertainty of ET measurements in humid conditions during and after rainfall events (Kang et al., 2018; Van Dijk et al., 2015), such analysis was not possible to conduct from the data. We note that IAV and trend of ET (Figures 2d and 3d) can also be markedly affected by such uncertainties, particularly in late autumn and wintertime when the contribution of non‐stomatal water fluxes is the greatest.

Leaf‐area growth was identified as the main driver of increasing GPP and NEP and clearly dominates over the direct $c_{a}$ effect (Figures 4a and 5a,e). This is in accordance with ecosystem model simulations of recent productivity trends in different (Li et al., 2018) but opposite to a FluxNet temperate and boreal forest site synthesis (Fernández‐Martínez et al., 2017) that reported no effect of LAI. At first glance, our finding seems to also be counter to the earlier finding from a thinning study at the same site (Vesala et al., 2005). In their study, one pre‐ (2001) and post‐thinning (2002) years were compared but no response of carbon or water fluxes to ca. 20% reduction in LAI was found. This was attributed to compensatory increase of ground vegetation photosynthesis and forest floor ET. Our model scenarios, however, suggest that favorable hydroclimatic conditions likely resulted in significantly larger GPP in 2002 than in 2001 (Figure S10). This underscores the need for long flux records and/or paired flux tower setups to “unpack” the LAI effects from IAV of hydrometeorological drivers and may well explain why thinning has been observed to either decrease ecosystem GPP and NEP (Lindroth et al., 2018; Misson et al., 2005) or show minor or non‐significant changes (Saunders et al., 2012; Vesala et al., 2005; Wilkinson et al., 2016). Moreover, the nonlinear response of ecosystem fluxes and WUE to LAI changes (Figure 5e and g) indicates the short‐term effects likely depend both on initial stand structure and thinning intensity.

### 4.3 Literature trends of boreal and temperate forest carbon and water exchange

Recent decades increase in global and boreal NEP appears to be not in dispute yet its magnitude and driving mechanisms remain a subject of inquiry (Fu et al., 2017; Haverd et al., 2020). Considering the length of FluxNet records, it is somewhat surprising that only a few studies have analyzed decadal or longer timeseries in detail for boreal and temperate forest carbon and water use trends.

One study in a Spruce forest in Germany found no trend in NEP, component fluxes or ET during a 10‐year period of flux measurements (Grunwald & Bernhofer, 2007). The 13‐year record from a Danish Beech forest showed increasing trend in annual NEP (+23 gC $m^{‐2}$ $a^{‐1}$, +14.6% $a^{‐1}$ of the long‐term average) and GPP (+29 gC $m^{‐2}$ $a^{‐1}$, or +1.7% $a^{‐1}$) (Pilegaard et al., 2011). That trend was primarily attributed to both longer CUP (+1.9 d $a^{‐1}$) and increase of ecosystem peak growing season photosynthetic capacity (+1.1% $a^{‐1}$). The latter trend magnitude is similar to what was found here (Figure S5), but the underlying causes must differ as Pilegaard et al. (2011) found no changes in ecosystem peak LAI.

Recently, a 19‐year long timeseries in boreal Black spruce forest in Canada was analyzed for trends in fluxes and resource use efficiencies (Liu et al., 2019). Contrary to our results, they found decreasing annual carbon sink (NEP −2.8 gC $m^{‐2}$ $a^{‐1}$, or −7.6% $a^{‐1}$), as the positive trend in $R_{e}$ (+7.8 gC $m^{‐2}$ $a^{‐1}$, or +1.1% $a^{‐1}$) exceeded that of GPP (+5.8 gC $m^{‐2}$ $a^{‐1}$, or +0.7% $a^{‐1}$). Following the GPP trend, annual LUE (+0.4% $a^{‐1}$) and WUE (+0.8% $a^{‐1}$) increased, the latter occurring as no change in ET was found. The magnitude of GPP trend and lack of change in ET agree with the findings here. Their annual LUE and WUE trends are also similar to what was observed here in favorable conditions during the core growing season (Figure S7).

Liu et al. (2019) attributed the $R_{e}$ trend to increasing autotrophic respiration ($R_{a}$) that lead the ecosystem carbon use efficiency (CUE = 1 − $R_{a}$/GPP) to decrease. We did not explicitly estimate, $R_{a}$ but a comparison of observed $R_{e}$ and GPP trends suggests annual CUE must either have increased, or heterotrophic respiration ($R_{h}$) decreased during the study period (Suppl. S8). In the absence of strong trends in environmental forcing, one could presume upper bound for $R_{h}$ trend is set by trend in litterfall. In Suppl. S8, we assumed litterfall changes proportionally with the observed development of $LAI_{a}$ and run a Yasso07 soil carbon model (Tuomi et al., 2009) to predict the resulting change in.$R_{h}$ When run with fixed climatic input, the model suggest $\delta R_{h}$ < 30 gC $m^{‐2}$ (or ca. 1.7 gC $m^{‐2}$ $a^{‐1}$), including a transient 50 gC $m^{‐2}\;a^{‐1}$ increase in $R_{h}$ following thinning in 2002 (Figure S13, Vesala et al., 2005). Furthermore, assuming time‐constant CUE, we can write. $\delta R_{e}=\left(1‐CUE\right)\delta GPP+\delta R_{h}$ Setting δGPP 120–170 over the 2001–2017 period (Table 2), yields $\delta R_{a}:$ 48–118 gC $m^{‐2}$ (or 2.8–7.0 gC $m^{‐2}$ $a^{‐1}$) when CUE is in range 0.3–0.6 typical for boreal forests (de Lucia et al., 2007; Goulden et al., 2011; Ilvesniemi et al., 2009; Ryan et al., 1997). This trend would alone be 2–4 times stronger than observed trend in annual $R_{e}$ (Tables 1 and 2).

Finzi et al. (2020) thoroughly analyzed temperate deciduous Harvard Forest carbon balance trends, IAV and regulating factors. Two decades of EC measurements indicated increasing annual NEP (+2.3% $a^{‐1}$ of the long‐term average), GPP (+1.5%$a^{‐1}$) and $R_{e}$ (+1.3%$a^{‐1}$); a particularly strong increase in NEP, GPP and WUE occurred from 1998 to 2008. They observed very strong IAV of carbon and water fluxes and suggested that canopy and leaf‐level trait plasticity such as IAV of LAI and $V_{\mathrm{cmax}}$ strongly contributes to IAV in addition to hydrometeorological factors. Drivers of such trait dynamics and its contribution to flux trends and IAV are far from understood and also omitted in our model scenarios. It was concluded that the increasing carbon sink and WUE was due to multiple, co‐occurring factors, including phenological changes, longer growing seasons (<1 d$a^{‐1}$), improved nutrient availability—and increasing $c_{a}$ (Finzi et al., 2020).

Fernández‐Martínez et al. (2017) found, on average, 1.0% $a^{‐1}$ annual increase in NEP and GPP. Using a statistical modeling, this was primarily attributed to direct $c_{a}$ effect and decreasing sulphur deposition positively affecting GPP and $R_{e}$. They found no trends in climatic drivers or LAI, and thus no contribution to carbon exchange. Wang et al. (2018) compared intrinsic WUE in 26 broadleaved and evergreen coniferous forests, mostly same sites as included in Fernández‐Martínez et al. (2017). They found average IWUE trend in deciduous (+1.93% $a^{‐1}$, marginally significant trend at 4 of the 11 individual sites) forests was twice that of coniferous (+0.85% $a^{‐1}$, 1 of the 15 sites) forests during the peak growing season. This resulted from concurrent increase in GPP (decid. +0.51% $a^{‐1}$, 2 sites; conif. +0.72% $a^{‐1}$, 3 sites) and constant or decreasing ET (decid. −0.09, no sites; conif. −0.6% $a^{‐1}$, 3 sites). Similar to our results (Figure 2f), the inter‐annual changes in IWUE were positively and $G_{s}$ negatively associated with those of D, and IWUE decreased with increasing PAR and $T_{a}$. Our results support their finding that environmental variability dominates IAV that is much stronger than the modest and highly scattered long‐term flux and WUE trends.

### 4.4 Challenges in detecting flux trends and their drivers

The analysis here and a meta‐analysis from the literature shows multiple factors simultaneously affect boreal forests productivity and water use trends across sites (Fernández‐Martínez et al., 2017; Keenan et al., 2013; Wang et al., 2018). The primary controls of IAV and trends vary seasonally and depend on the timescale considered (Figure 2). The processes underlying instantaneous carbon and water fluxes are non‐linear with respect to their environmental drivers and the effect of hydrometeorological variability tends to be ameliorated when fluxes are integrated to longer timescales. As the same seasonal or annual balances can arise from numerous “pathways,” statistical modeling of the subtle decadal trends using seasonal / annual climatic averages may not pinpoint correct drivers of the trends or their contribution.

To bypass such challenges, the trends in NEP, GPP, and water use were analyzed across averaging periods and conditions complemented with process‐based ecosystem model scenarios to explain the observed 2001–2017 growing‐season integrated carbon and water flux trends (Figure 3). Comparing data and prognostic simulations, where measured time series of potential drivers were sequentially added and their impact on modeled trends analyzed, allows testing alternative hypotheses and identifying deviations between “expected” and observed ecosystem response (Haverd et al., 2020; Lee et al., 2018; Yue et al., 2015). When performed at the site level, such comparisons can also reveal discontinuities and biases in long‐term flux data, which are difficult to detect with time series analysis only. For instance, the model‐data comparison (Figures S8 and S9) suggest time‐dependent biases in EC‐based ET can be linked to the performance of EC system in detecting evaporation of intercepted rainfall (Kang et al., 2018; Van Dijk et al., 2015). Moreover, annual and seasonal GPP and $R_{e}$ trends obtained from different flux partitioning methods differed by factor of >1.5 (Figure 3). If being a more than a site‐specific observation, the flux partitioning uncertainties may affect interpreting the causal mechanisms underlying long‐term NEP, GPP, and WUE changes (Lavergne et al., 2019).

The model results suggest ecosystem $c_{a}$ response is weaker than that observed at the leaf level, similar to what was recently reported for EucFACE (Yang et al., 2020). For the ca. 10% increase in $c_{a}$ during 2001–2017 here, the direct effect on GPP was +3–4% depending on the growing season. For annual average GPP 1110–1180 $m^{‐2}$ $a^{‐1}$ observed in the study here (Table 2), the corresponding direct $c_{a}$ effect would be 2–3 gC $m^{‐2}$ $a^{‐1}$ and LAI‐effect 4–5 gC $m^{‐2}$ $a^{‐1}$. Making the conservative estimate that the IAV (40–60 gC $m^{‐2}$ $a^{‐1}$) represents the random uncertainty of GPP, we followed Weatherhead et al. (1998) and Baldocchi et al. (2018) approaches and estimated the statistical detection limit (*p* < .05) for trend in our 17‐year time series to be 2.5–3.0 gC $m^{‐2}$ $a^{‐1}$. Thus, the direct $c_{a}$ effect may be weaker than what can be actually detected from the flux data. The model simulations further suggest that the apparent proportionality of ecosystem GPP trend to $c_{a}$ arises as increasing LAI compensates for the decreasing mean leaf stomatal conductance. Such long‐term physiological adjustments cannot be detected unambiguously from flux data alone as they require combination of theoretical analysis (Cernusak et al., 2019; Li et al., 2018; Yang et al., 2020), and detailed information on LAI development and management history.

The results here support prior suggestions that understanding the processes and drivers of LAI dynamics are crucial to assess the responses of ecosystem carbon cycle to changes in $c_{a}$ and hydrometeorological variability (Li et al., 2018; Yue et al., 2015). This is particularly important for Fennoscandia and in parts of boreal Russia and Canada, where significant fraction of forests are subject to management. For instance in Finland, <85% of the forests are managed and the forest management history and current practices such as clear‐cutting and different intensity thinnings, dictate the productivity changes, LAI dynamics and forest structure over any climatic trends (e.g. Henttonen et al., 2017). This is the case also for the Hyytiälä forest, which was sown in 1962 and thinned in 2002 following standard practices in even‐aged clear‐cut forestry. Thinning reduces among‐tree resource competition and improves the productivity and growth of the remaining trees. The improved understory light conditions further enable spruce and deciduous undergrowth to develop below the main canopy. On ecosystem scale, this was seen primarily as increasing spruce and deciduous LAI with stand age (Figure S4), a typical behaviour on medium‐fertility sites in the boreal zone.

The results here further showed that increasing canopy roughness and concomitant shrinking of flux footprint imposes additional challenges to estimate footprint‐weighted LAI and species composition trends at site level. The effects of such small‐scale heterogeneities, occurring often at scales beyond the resolution of current LAI products, should be further explored whenever interpreting long‐term trends from forest FluxNet sites (Foken et al., 2021). In the Nordic countries, emerging high‐resolution (sub 25 m) biomass and vegetation data products that combine data from national forest inventory plots and remote sensing (Kangas et al., 2018) can provide an interesting opportunity for such analyses, as well as for benchmarking the global LAI products (Härkönen et al., 2015; Zhu et al., 2013b).

## 5 SUMMARY

We analyzed 17 years (2001–2017) of EC flux data from a managed boreal coniferous‐dominated forest for IAV and trends in carbon balance and ET. We found the forest was a consistent annual carbon sink (mean annual NEP 252 gC $m^{‐2}\;a^{‐1}$), and the sink strength increased 100–110 gC $m^{‐2}\;a^{‐1}$ (or ca. 50% of the initial level) during the period. This increase was attributed to enhanced GPP and occurred without significant alterations to the water cycle as ET did not change. Increase of GPP and NEP outside the main growing season (May–September) contributed ca. 1/3 and 1/4 of the annual trend, respectively. A marginally significant advancement in the start of the annual CUP was observed. Meteorological factors act as main drivers of the physiological adjustments ($g_{s}$, $c_{i}/c_{a}$, Equation 2) that regulate diurnal, seasonal, and IAV. They did not, however, explain any of the long‐term trends in NEP, GPP, or resource use efficiencies.

The observed growing season GPP trend was roughly proportional to $c_{a}$ trend, and even stronger in the most favorable conditions during the peak growing season. Using a multilayer ecosystem model we proposed that direct $c_{a}$ fertilization effect depends strongly on environmental conditions and its magnitude decreases when moving from leaf to ecosystem scale. As a result, only 30–40% of the observed 2001–2017 GPP increase may be attributed to $c_{a}$. The observed trends (or their lack of) in GPP, LUE and ecosystem water use were similar to what was expected based on the established theory of the coupling between leaf and ecosystem carbon, water and energy exchange when both $c_{a}$ and LAI increase was accounted for in the simulations. The canopy average leaf stomatal conductance decreases both as response to increasing $c_{a}$ (direct fertilization effect) and LAI (increasing light limitations). However, at ecosystem scale, the increasing LAI compensates for these physiological adjustments causing the apparent proportionality between observed GPP and $c_{a}$ trends. This compensatory mechanism also explains why ecosystem surface conductance, ET and $C_{i}/C_{a}$ remained conservative with respect to both LAI and $c_{a}$ variations. The results emphasize that comparing or interpreting $c_{a}$ effects across studies and biomes must pay increasing attention to both spatial (leaf to ecosystem) and temporal (seasonal to inter‐annual) scales.

Based on the model‐assisted analysis of long‐term flux data, we conclude that LAI development—not the increasing atmospheric ${\mathrm{CO}}_{2}$—was the primary mechanism explaining the increasing carbon sink of a mid‐rotation boreal coniferous forest. In managed boreal forests, LAI dynamics is strongly driven by management history and stand age, which should be better accounted for when interpreting long‐term trends from FluxNet data. Attributing trends in carbon and water fluxes to their physical and physiological drivers was challenged by strong IAV and uncertainty of LAI and species composition changes due to the dynamic flux footprint of EC measurements. These findings merit further analysis to better constrain mechanisms of increasing terrestrial carbon uptake.

## Supporting information

Supplementary Material

## Data Availability

The data from Hyytiälä (FI‐Hyy) site that support the findings of this study are openly available in following repositories. EC‐data: http://urn.fi/urn:nbn:fi:att:af0b5d17‐6630‐43a6‐acf8‐223064a8bd82; Meteorological and soil data: http://urn.fi/urn:nbn:fi:att:a8e81c0e‐2838‐4df4‐9589‐74a4240138f8; Site characteristics and vegetation: https://doi.org/10.5281/zenodo.5909681. The gap‐filled dataset used in this study, including model forcing files for 2001–2017, and the APES model source code (Python 3.7) are available by request from the corresponding author.
