# Supplementary material for: Does growing atmospheric CO2 explain increasing carbon sink in a boreal coniferous forest?
Source: Glob Chang Biol. 2022 Feb 22;28(9):2910–29. doi: 10.1111/gcb.16117 (PMC9544622; doi:10.1111/gcb.16117)
Supplement: Supplementary file 1 — Supplementary Material [file GCB-28-2910-s001.pdf]

Supplementary material: Does growing atmospheric CO_2 explain increasing carbon sink in a boreal coniferous forest?

Samuli Launiainen[1,*], Gabriel G. Katul[2], Kersti Leppä[1,+], Pasi Kolari[3,+], Toprak Aslan[3,+], Tiia Grönholm[4,+], Lauri Korhonen[5,+], Ivan Mammarella[3,+] and Timo Vesala[3,6,7]

[1]Natural Resources Institute Finland, Latokartanonkaari 9, FI-00790, Helsinki, Finland

[2]Duke University, Department of Civil and Environmental Engineering, Durham, NC, 27708-0328, USA

[3]Institute for Atmospheric and Earth System Research/Physics, Faculty of Science, University of Helsinki, Finland

[4]Finnish Meteorological Institute, Erik Palmenin aukio 1, FI-00560, Helsinki, Finland

[5]University of Eastern Finland, Joensuu, Finland

[6]Institute for Atmospheric and Earth System Research/Forest Sciences, Faculty of Agriculture and Forestry, University of Helsinki, Finland

[7]Yugra State University, 628012, Khanty-Mansiysk, Russia

[*]samuli.launiainen@luke.fi

[+]these authors contributed equally to this work

January 30, 2022

S1 Flux measurements and data processing

The 1/2 h NEP, ET and turbulent fluxes of sensible heat and momentum were measured using the eddy covariance (EC) technique in accordance with FluxNet guidelines (Aubinet et al., 2012). The measurement system included an ultrasonic anemometer (Solent

Research 1012R2, Gill Instruments Ltd, Lymington, Hampshire, England) for measuring wind velocity components and virtual temperature, and a closed-path infrared gas analyzer (LI-6262, LI-COR Biosciences, Lincoln, NE) for measuring the CO_2 and H_2O mole fractions in air. The measurements were initiated in 1996 (Markkanen et al., 2001), however in 1/1999 - 6/2000 the EC measurement height was 46 m. Sample line heating was installed in 2001 after which the quality of water vapor fluxes improved. From 4/2018 the EC setup was replaced with the one following ICOS standards, and the measurement height was increased to 27 m. For consistency, we focus here on the 17 year period from 2001–2017 when fluxes were measured at the same 23.3 m height.

The fluxes were computed using the EddyUH software (Mammarella et al., 2016) following the standard approach for EC data processing Aubinet et al. (2012). Prior to flux calculations, raw data despiking, conversion of CO_2 and H_2O from wet to dry mole fraction and double rotation of the coordinate system reporting the velocity components were performed. The time delay between the vertical velocity and CO_2 and H_2O was derived for each 1/2 h interval by maximizing their respective cross-correlation functions. A constant search window was used for CO_2 time delay estimation, whereas for H_2O the lag window size was varied as a function of relative humidity (RH) (Mammarella et al., 2016; Nordbo et al., 2011).

The turbulent fluxes were corrected for low frequency underestimation using the theoretical transfer function for block averaging (Rannik and Vesala, 1999). High-frequency response correction for momentum and sensible heat flux was performed using theoretical transfer function (Aubinet et al., 2012), while experimental transfer functions were used for CO_2 and H_2O fluxes. These were estimated as the ratio of the measured cospectrum of respective scalar and kinematic heat flux. A first-order Lorentzian function was fitted to the measured transfer function to retrieve the low-pass

filter time constant (Eugster and Senn, 1995) . To account for sorption/desorption effects on the sampling line (Nordbo et al., 2014), the transfer function for H_2O was calculated for different RH classes and for different years (Mammarella et al., 2009). The low pass filter time constant for CO_2 was 0.25 s, while for H_2O it varied between 0.3 s and 8 s depending on RH and age of the sampling line.

The fluxes were further quality screened by applying limits for 1/2 h skewness (-2 < Sk < 2) and kurtosis (1 < Ku < 8) of vertical velocity and scalar mixing ratios. In addition, data were rejected if the second coordinate rotation angle was outside the range $\pm$ 15°. The flux stationarity test was not used. The measured NEP, ET and sensible heat fluxes were finally corrected for storage changes below the measurement height using mean 1/2 h air temperature and mean concentrations sampled at several heights below the EC setup (Kolari et al., 2009; Launiainen, 2010).

S2 Effect of flux partitioning methods on GPP and R_e trends

To explore if flux partitioning affects annual and seasonal balances and trends, we compared the standard FluxNet nighttime and daytime approaches (Wutzler et al., 2018) and a site specific method (Kolari et al., 2009). Timeseries of annual GPP and R_e, and the relation between different partitioning results are shown in Fig. S1.

Site-specific method

NEP values measured under low turbulence were excluded based on a friction velocity (u_*) threshold 0.3 m s^{-1}. The NEP was partitioned into GPP and R_e using the ecosystem light-response curve. Thus curve was characterized by fitting a non-rectangular

hyperbola to NEP averaged across PAR bins (Kolari et al., 2009)

$$NEP = \frac{\alpha\, PAR + P_{max} - \sqrt{(\alpha\, PAR + P_{max})^2 - 4\theta\, PAR P_{max}}}{2\theta} - R_e, \tag{S1}$$

where α (-) is the initial slope, P_{max} (μmol $m^{-2}s^{-1}$) is light-saturated NEP and θ (-) a shape parameter. Response to instantaneous T_a was introduced to bring GPP to zero at freezing temperatures (Kolari et al., 2014)

$$f(T_a) = -\frac{1}{1 + exp(2\,(T_0 - T_a))}, \tag{S2}$$

where T_0 is a parameter (inflection point) and T_a air temperature. The R_e was as exponential temperature function fitted to night time NEP

$$R_e = R_{ref}\, Q_{10}^{T/10}, \tag{S3}$$

where R_{ref} (μmol m^{-2} s^{-1}) is the base respiration rate at 10 °C and Q_{10} (-) the temperature sensitivity. The driving temperature T was taken as the mean of T_a at 17 m height and T_s at 2 cm depth in the mineral soil. The light-response parameters (eq. S1) were estimated for time periods of 11 days with 5 day overlap, whereas Q_{10} = 2.0 and θ = 0.75 were set based on data from the whole study period. When the turbulence criteria were met, GPP was calculated as the difference between modelled R_e and measured NEP. Missing or rejected NEP values were gap-filled using modelled GPP and R_e.

FluxNet -methods

We used the REddyProc online tool (Wutzler et al., 2018). In the nighttime approach (Reichstein et al., 2005), flux partitioning is based on parameterizing the R_e model using

night-time NEE similar to eq. S3

$$R_e = R_{ref}\, e^{E_0\,(1/(T_{ref}-T_0)-1/(T-T_0))}, \tag{S4}$$

where R_{ref} is base respiration rate at reference temperature T_{ref} and T_0 = -46.02°C a fixed parameter. The activation energy E_0 is first estimated as mean of individual values in 15-day windows, and R_{ref} then re-computed in 7-day windows with 3-day overlap and linearly interpolated between the midpoints of the windows. The GPP is calculated as the difference between modelled R_e and NEP.

In the daytime approach (Lasslop et al., 2010), NEP is modelled directly as

$$NEP = \frac{\alpha_l\,\beta\,R_g}{\alpha_l\,R_g + \beta} + R_e, \tag{S5}$$

where R_g is global radiation and α_l and β light response parameters. The parameter estimation is done in moving windows (Wutzler et al., 2018). As both GPP and R_e are derived from eq. S5 fitted to NEP data, their sum does not add up exactly to observed NEP contrary to the site-specific or nighttime methods. The nighttime approach was applied using both T_a at 17 m height and soil temperature T_s at 2cm depth in mineral soil as driving variable, while T_a was used in daytime method.

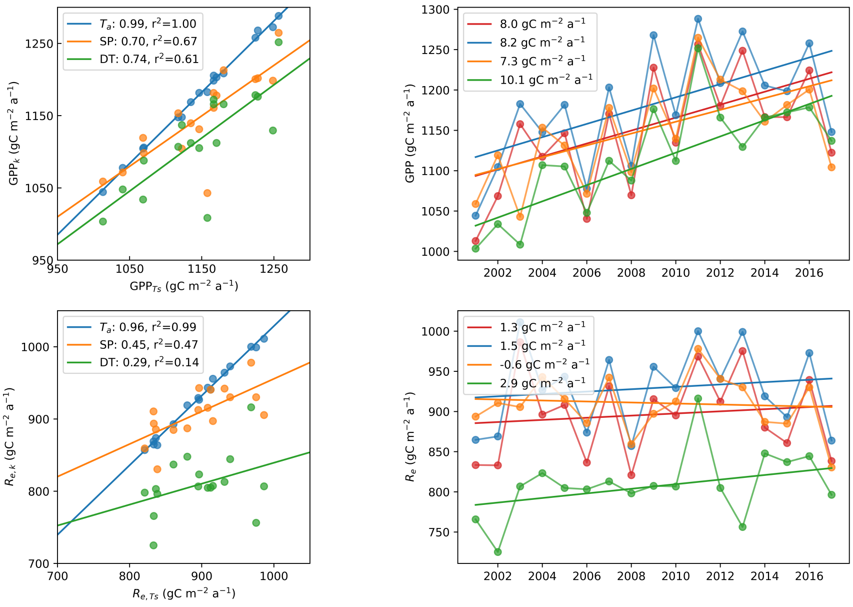

Figure S1: Annual GPP and R_e from different flux partitioning schemes. The scatterplot shows slope and r^2 of linear regression using soil-temperature -based nighttime method as the reference (x-axis). T_a air-temperature, SP site specific, DT daytime method.

S3 Leaf-area index and footprint changes

The leaf-area index (LAI, $m^2 m^{-2}$) was not consistently measured throughout the study period. Instead, we used both an allometric method (LAI_a) and canopy light interception data (LAI_o) to estimate trend in tree stand LAI (Fig. S2a). In the allometric method, stand inventory results in 1997–2015, representing the distribution of diameter at breast height and tree height per species, were first converted to needle/leaf mass per ground area using biomass equations (Marklund, 1988; Repola et al., 2009). Specific leaf areas (SLA) of 6.8, 4.7 and 12.0 m^2 kg^{-1} for Scots pine, Norway spruce and deciduous species (Härkönen et al., 2015), were then used to convert respective leaf masses to LAI_a. The tree inventory results and total LAI_a represent the average over circular area with radius of 200 m centered on the main mast. Tree sampling protocol is detailed in Ilvesniemi et al. (2009).

The LAI_o was estimated from PAR measurements made at 0.6 m above the forest floor at one location since autumn 2003, and at two additional locations since 2010. At each location, 1/2 h averages of 4 or 5 PAR sensors (Li-Cor Li-190, Apogee SQ100) installed on 3-m-long horizontal booms were used. The LAI_o was determined from the ratio of below canopy (Q_b) to above-canopy Q_0 PAR as

$$LAI_o = \frac{1}{k} ln \left(\frac{Q_b}{Q_0} \right), \tag{S6}$$

where k (-) is the extinction coefficient. Only data from overcast conditions (diffuse to total PAR ratio > 0.85) were used, and peak LAI_o values (in August) selected for each year. Compared to LAI_a, the optically derived LAI and its development represents the part of the stand closest to the main mast (Fig. S3).

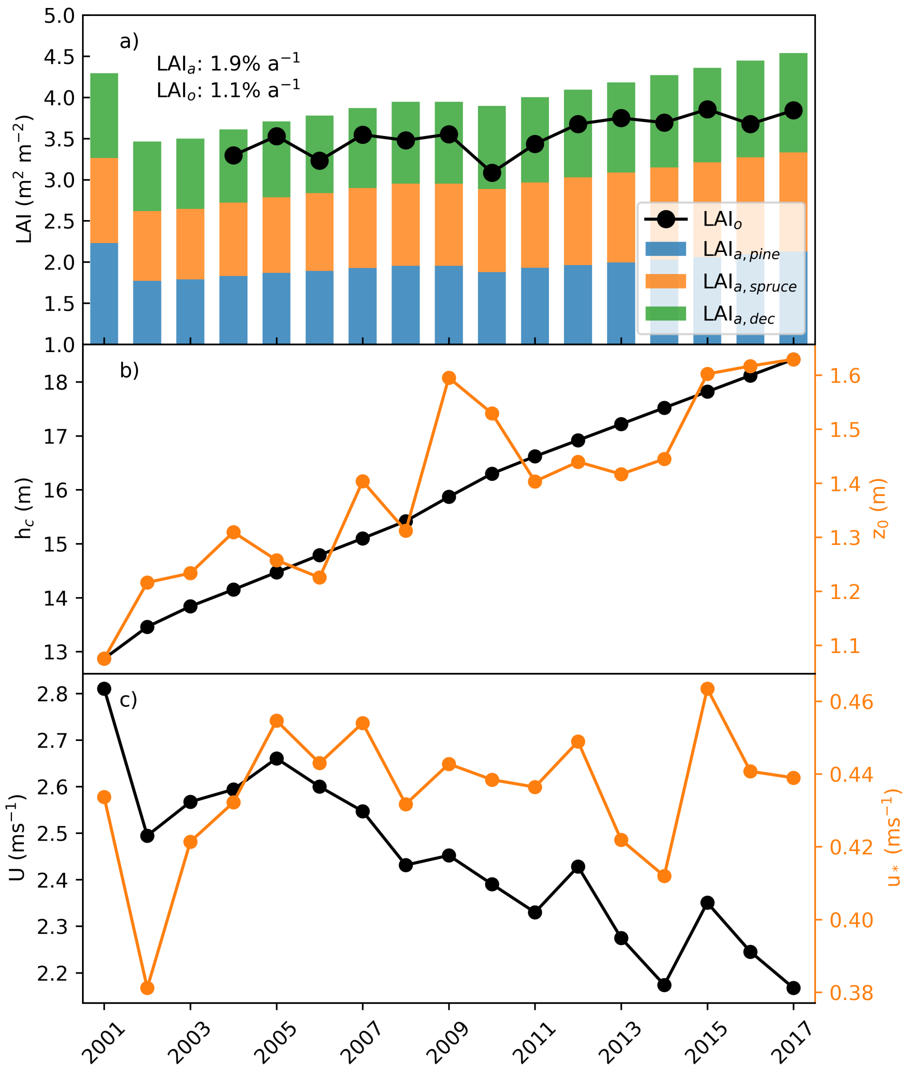

Figure S2: Leaf-area index (LAI) per species estimated from tree-inventories (LAI_a) and from canopy light interception data (LAI_o). The stand was partially thinned in winter 2002 (Vesala et al., 2005) and small snow damages occurred in 2010 (a), mean canopy height (h_c) and roughness length for momentum (z_0) (b), and mean May-Sept wind speed (U) and friction velocity (u_*) at 23 m height (c).

We used Kljun et al. (2015) model to address how stand growth has changed the EC flux footprint during the study period. The increase of dominant stand height from ∼13 m in 2001 to ∼18 m in 2018 (Fig. S2b) did not affect u_* -threshold used for filtering low-turbulence conditions from the data. However, footprint model results indicate stand growth and concomitant increase in momentum roughness length has strongly 'compressed' the flux source area. Fig. S3a shows the respective boundaries of the 80% footprints for years 2001, 2006, 2011 and 2016 (in decreasing distance from the EC tower located at the origin) embedded on top of a LiDAR-based LAI-raster at 10 m resolution from year 2011. The raster was obtained by linear regression analysis based on n = 76 field plots with an optical LAI estimate and airborne LiDAR data from the surrounding area (Bruguiére, 2020). In addition, Fig. S3b-d show the relative stem volume of pine, spruce and deciduous tree species, respectively, illustrating the spatially heterogeneous stand distribution. The species stem volume raster at 16x16 m resolution was provided by the Finnish Forest Centre open forest data (`https://www.metsakeskus.fi/fi/avoin-metsa-ja-luontotieto`). The area around the tower is dominated by pine in contrast to spruce, which is densely spread out at the southeast direction.

Understanding how footprint change and stand growth have jointly altered LAI and its partitioning into species during the study period is of importance in such heterogeneous forest. Unfortunately, we could not directly monitor those changes due to the absence of comparable LAI maps yearly obtained throughout the study period. Therefore, we made such comparison using the only available LAI map, which is from 2011, assuming that the spatial distribution of LAI and species composition do not change in time and follow that observed in 2011 as the footprint inter-annually changes as shown in Fig. S3a. For this, we computed the effective (average) LAI inside the 80% footprint

contour for each year. The same was done for the species composition by calculating the normalized stem volume. In addition, we calculated the yearly normalized footprint area, quantitatively representing the change with respect to the footprint area in 2001 (20.5 ha). The results are shown in Fig. S4. The source area shrank ca. 70%, which increased the stem volume of pine, decreased spruce and had negligible change in deciduous cover fraction within the footprint. The decrease of effective LAI by < 10% results from densest spruce-dominated parts being left outside the footprint. This LAI change would have been smaller than the effect of stand growth on both LAI_a and LAI_o (Fig. S2). The allometrically derived LAI_a suggest a growth rate of ca. 1.9% a^{-1} since 2003, with the growth of spruce and deciduous tree LAI (more abundant at larger distances from the EC tower) being faster than that of Scots pine. LAI_o, which was estimated from light attenuation in the pine-dominated part close the EC tower, increased at a slower rate (1.1% a^{-1}) resembling the growth rate of pine in LAI_a (Fig. S2). Putting these findings together, it is likely that the LAI_a trend, obtained from stand inventories made at fixed locations, is an overestimate of 'true' leaf-area changes within the EC-footprint. We include LAI trend uncertainty (using both LAI_a and LAI_o) into our model scenarios to explore its impact on the conclusions.

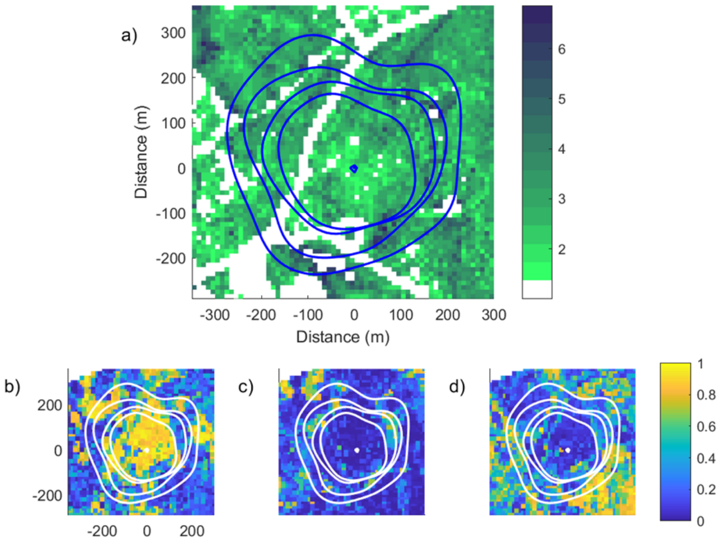

Figure S3: Spatial patterns of LAI and changing footprint are shown in (a). The LAI-raster with 10 m resolution is derived from airborne-LiDAR (2011). The blue contours show the 80% footprint boundaries for 2001, 2006, 2011 and 2016 (in descending order). The EC tower is located at the origin. Relative stem volume (species-specific stem volume divided by total stem volume) are shown in (b), (c) and (d), representing pine, deciduous and spruce tree species, respectively. The white contours show the footprint boundaries similar to (a).

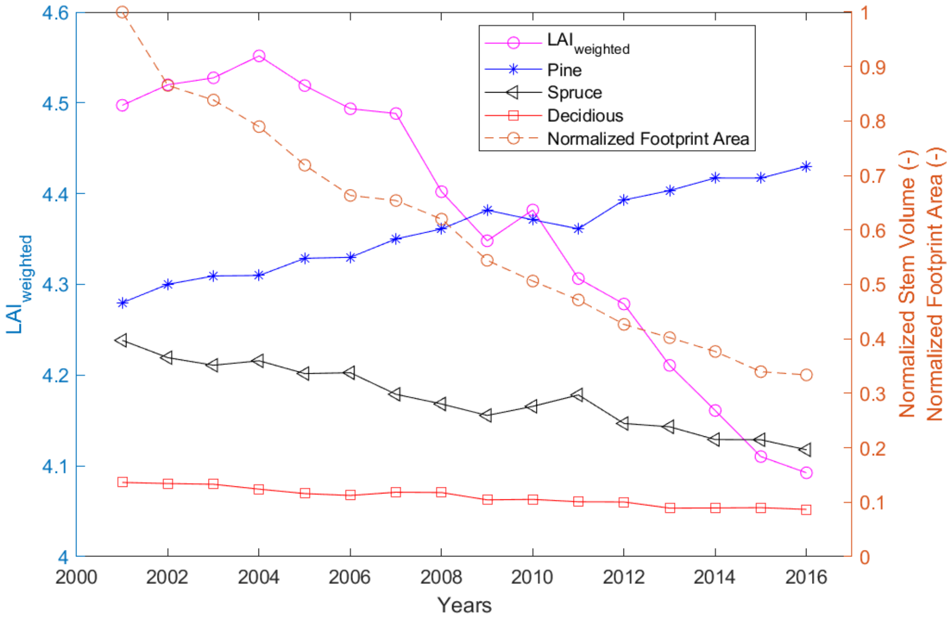

Figure S4: The change of footprint area and its effect on average LAI, i.e. $\mathrm{LAI}_{weighted}$, and species composition within the 80% footprint. The source area shrinks ca. 70% compared to 2001 (20.5 ha). The LAI shows ca. 10% decrease from 2004 to 2016, and the contribution of Scots pine to total stem volume increases in case when forest growth is not accounted for and spatial patterns of LAI and stem volume assumed to remain as in 2011.

S4 Carbon uptake period and thermal growing season

We retrieved start and end dates of carbon uptake period (CUP) following recommended procedures from other studies (Zhu et al., 2013). Briefly, daily NEP is first smoothed with a 15-day running mean filter. To detect the start (SCU) of carbon uptake, a 10-day subset during which the first 5 elements of smoothed NEP are smaller than zero and the last 5 elements greater than zero are selected and SCU estimated as the zero intersection of linear fit to the daily NEP. The end of carbon uptake (ECU) was determined similarly as the transition date from positive to negative daily NEP.

Thermal growing season onset and end was determined as last day of first (last) 6-day period in spring (autumn) during which daily mean T_a was higher (lower) than +5°C (Linderholm, 2006).

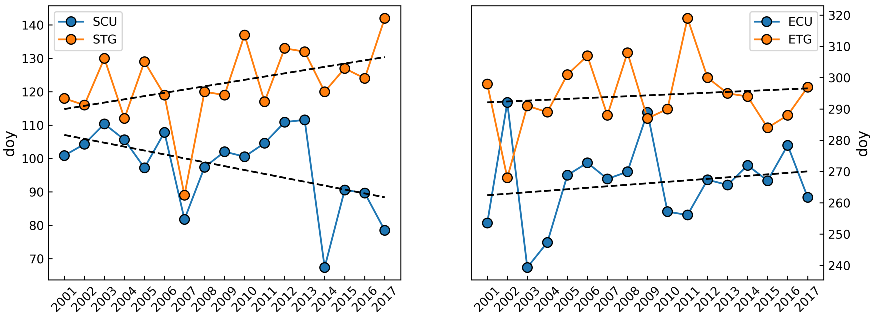

Figure S5: Carbon uptake start (SCU) and end (ECU) dates defined from net ecosystem productivity, as well as thermal growing season start (STG) and end dates (ETG). Trends are statistically insignificant (p>0.05)

S5 Light-response and big-leaf parameters during the core growing season

We evaluated ecosystem light-response and water use in the core growing season using measured data (i.e. non-gapfilled). We considered 1 July to 10 August as a period when ecosystem properties are stationary in terms of seasonal cycle of LAI, phenology and soil processes. To ensure storage fluxes did not affect the analysis, u_* > 0.30 m s^{-1} threshold was applied also for previous 1/2 h period. Only data measured in dry-canopy conditions (no rain in the last 12 h), sufficiently moist soils (θ > 0.15 m^3 m^{-3} and minimum θ preceding the period >0.11 m^3 m^{-3}) were considered to minimize non-stomatal water sources and to exclude possible carry-over effects of drought, respectively. The latter criteria removed most of 2006 data. To standardize for environmental conditions, data was further clustered into PAR classes, in which constrained T_a and RH ranges were applied (Table S5).

Table 1: PAR-classes and their T_a and RH ranges used to bin-average data

**PAR (μmol m^{-2}s^{-1})**	**<20**	**20–200**	**200–400**	**400–700**	**700–1000**	**1000–1600**
T_a (°C)	10–16	12–18	13–20	14–22	16–24	18–26
RH (%)	60–100	50–100	50–90	40–80	40–70	40–70

The ecosystem light-response curve was characterized by a non-rectangular hyperbola (eq. S1) to NEP first averaged to PAR bins. The base rate of ecosystem respiration at 10 °C ($R_{ref,10}$) was obtained from nighttime (PAR < 20 μmol m^{-2} s^{-1}) as $R_e = R_{ref,10}\, Q_{10}^{(T/10}$, where $Q_{10} = 2.0$ and mean of T_a and T_s at 2 cm depth in the mineral soil was used as driving temperature. The results show ecosystem level photosynthesis has increased at high light (here shown as P_{1200}) but not at low PAR as there was no change in α (Fig. S6). Neither did $R_{ref,10}$ show any trend. Inter-annual variability of both $R_{ref,10}$ and α was ± 15 % relative to their respective mean values.

Fig. S7 shows changes in GPP, resource use efficiencies and bulk ecosystem measures (G_s, c_i/c_a) during dry-canopy conditions in the core growing season (July 1st to Aug. 10th). The strongest trends in GPP (1.0 % a^{-1}) were found at PAR range 700–1000 μmol m^{-2} and at PAR >1000 μmol m^{-2} s^{-1}. At the former PAR regime also light use efficiency increased but the trends became insignificant at higher irradiance. As dry-canopy ET and G_s were not much altered, also water use efficiency increased, again mostly strongly at PAR range 700–1000 μ mol m^{-2} s^{-1}. No change in ecosystem c_i/c_a or surface conductance was found.

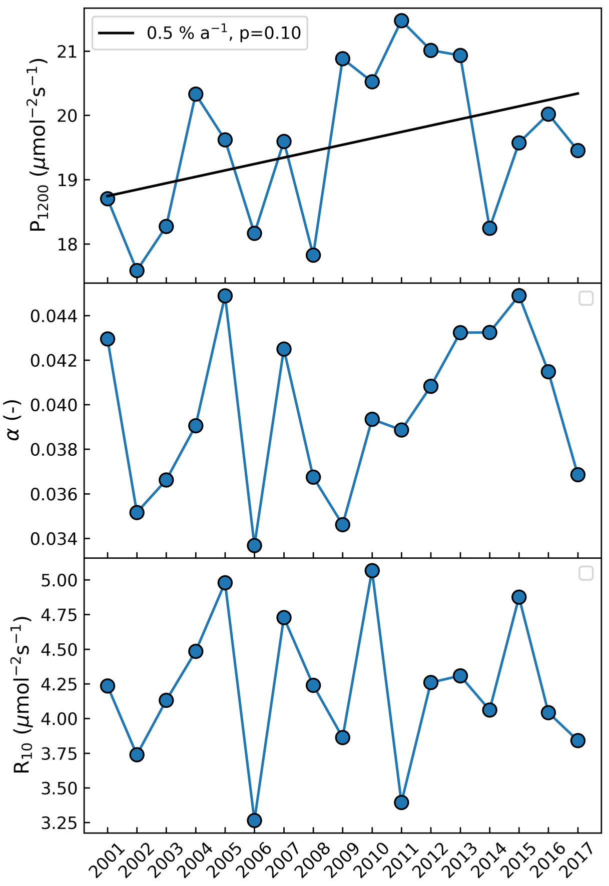

Figure S6: Changes in the ecosystem light response curve during the core growing season (1st in the July - Aug. 10th). Only dry-canopy conditions in absence of soil water limitations are included and the panels show NEP at 1200 μmol m^{-2}s^{-1} (NEP$_{1200}$), slope of light response (α) and base rate of ecosystem respiration (R$_{ref,10}$) (eq. S1).

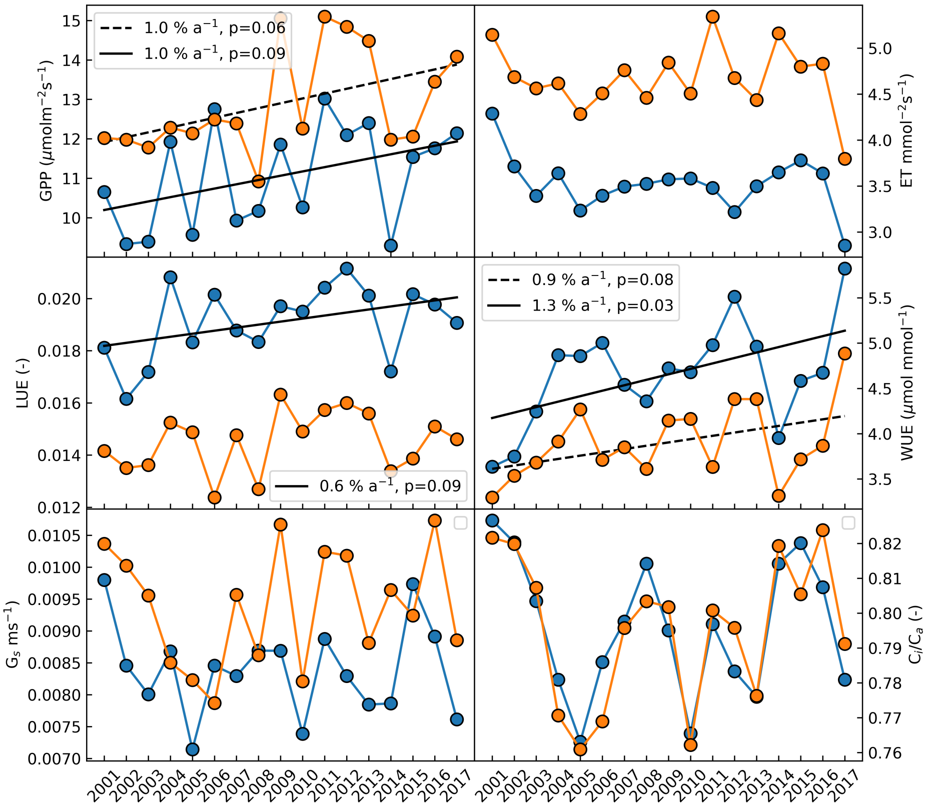

Figure S7: Mean Gross-primary productivity (GPP), evapotranspiration (ET), light use (LUE) and water use (WUE) efficiencies, ecosystem surface conductance (G_s) and effective leaf internal to external CO_2 (C_i/C_a) for the two highest PAR-classes (700–1000 (blue) and 1000–1600 μmol m^{-2}s^{-1} (orange) 1st in the July - Aug. 10th period. Only dry-canopy conditions in absence of soil water limitations are included. Marginally significant (p<0.1) trends are shown.

S6 APES model

Parameterization

The APES model was parameterized for the SMEAR II -site mainly following Launiainen et al. (2015) and Leppä et al. (2020). The former performed thorough test of the model against measured above and sub-canopy EC-fluxes, air-space scalar gradients and soil moisture and temperature profiles. We consider four vascular plant types: Scots pine & Norway spruce, deciduous trees and understory shrubs. Normalized leaf-area density (lad) profiles for the tree species were determined from tree inventory done in 2011 using crown distribution models (Tahvanainen and Forss, 2008) as in Leppä et al. (2020). Species-specific leaf traits were obtained from the literature, with exception of soil moisture response of stomatal parameter g_1 and V_{cmax25}, which was determined from continuous shoot/leaf gas-exchange chamber measurements at the study site (Launiainen et al., 2015).

The V_{cmax25} and g_1 (eq. 5) were adjusted as a non-linear function of plant available water (Keenan et al., 2010; Zhou et al., 2013) as

$$f_w = min\left[1.0, \left(\frac{Rew}{b_0}\right)^{b_1}\right], \quad \text{(S7)}$$

where $Rew = (\theta - \theta_r)/(\theta_s - \theta_r)$ is plant available water and b_i fitting parameters. The soil water content θ was taken from measured water content at ca. 5cm depth in the mineral soil and field capacity and residual water content set to θ_s=0.30 m^{3}m^{-3}, θ_r = 0.03 m^{3}m^{-3} based on soil type at the site. The b_i differ between g_1 and V_{cmax25} but in absence of species-specific data we used same drought response for all plant types.

Table 2: Parameters of pyAPES model for SMEAR II site.

Description	Parameter value	Source
**Canopy radiation parameters**		
Clumping coefficient (-)	0.7	Campbell and Norman (1998)
Leaf-angle distribution (-)	1.0 (spherical)	Campbell and Norman (1998)
Shoot PAR albedo (-)	0.1	Adjusted to match canopy albedo
Shoot NIR albedo (-)	0.39	Adjusted to match canopy albedo
Leaf emissivity (-)	0.98	Campbell and Norman (1998)
**Canopy flow parameters**		
Foliage drag coefficient (-)	0.15	Katul et al. (2004)
**Canopy interception parameters**		
Maximum interception storage for rainfall (mm)	0.2 × LAI	Watanabe and Mizutani (1996)
Leaf orientation factor (-)	0.5 (random)	Watanabe and Mizutani (1996)
**Plant type parameters for photosynthesis–stomatal conductance models**		
Carboxylation capacity V_{cmax} at 25°C (µmol m-2 s-1)[a]	V_{cmax25}	see Table 3
Electron transport capacity J_{max} at 25°C (µmol m-2 s-1)[a]	1.97 × V_{cmax25}	Kattge and Knorr (2007)
Leaf dark respiration rate rd at 25°C (µmol m-2 s-1)[a]	0.023 × V_{cmax25}	Launiainen et al. (2015)
Co-limitation parameter	0.95	Collatz et al. (1990)
Curvature of electron transport light response (-)	0.7	Launiainen et al. (2015)
Quantum yield parameter (mol mol-1)	0.2	Launiainen et al. (2015)
Stomatal model slope (-)	g_1	see Table 3
Residual conductance (mol m-2 s-1)	g_0	see Table 3
Drough response parameter b_0 for g_1 and V_{cmax25} (-)	0.39 and 0.31	
Drough response parameter b_1 for g_1 and V_{cmax25} (-)	0.83 and 3.0	

LAI = leaf area index; PAR = photosynthetically active radiation; NIR = near infrared radiation
[a] Temperature response curves of V_{cmax} and J_{max} are adopted from Kattge and Knorr (2007) and Medlyn et al. (2002), and of r_d from Launiainen et al. (2015).

Table 3: Plant type specific model parameters.

Description	Parameter value			
	**Pine**	**Spruce**	**Birch**	**Shrubs**
Maximum LAI, LAI$_{max}$ (m^2 m^{-2})[a]	0.43 × LAI$_{tot}$	0.21 × LAI$_{tot}$	0.21 × LAI$_{tot}$	0.15 × LAI$_{tot}$
Minimum LAI (m^2 m^{-2})[b]	0.8 × LAI$_{max}$	0.8 × LAI$_{max}$	0.1 × LAI$_{max}$	0.6 × LAI$_{max}$
Minimum value for seasonal cycle modifier (-)[c]	0.1	0.1	0.01	0.1
Characteristic leaf length scale (m)	0.02	0.02	0.05	0.05
Nitrogen attenuation coefficient (-)	0.5	0.5	0.5	0
V_{cmax25} (μ mol m^{-2} s^{-1})	50	50	40	40
Stomatal model slope g_1 (-)	2.8	2.8	5.2	5.2
Residual conductance g_0 (mol m^{-2} s^{-1})	0.001	0.001	0.005	0.005

LAI = leaf area index; V_{cmax25} = Carboxylation capacity at 25°C
[a]For canopy LAI (LAI$_{tot}$) see Fig. S2
[b]Seasonal development of LAI starts when the degree day sum (base temperature 5$\circ$C) exceeds 45 and reaches maturation at 250. Leaf senescence in autumn follows Launiainen et al. (2015).
[c]Seasonal cycle modifier for photosynthetic capacity is based on the delayed effect of temperature (Kolari et al., 2014; Launiainen et al., 2015).

Feasibility check against data

The comparison of model results against measured data are presented in Figs. S8 & S9. The results are shown for the model simulations accounting for both c_a fertilization and LAI_a. Excluding times with rain during past 12 h (Fig. S9) improves especially the fit between modelled and measured ET as times of rainfall interception and subsequent evaporation are excluded. We also observe how the model-data bias in ET including wet-canopy conditions varies across years, being largest in 2004 – 2005 & 2010 – 2011. We interpret this as potential underestimation of measured wet-canopy evaporation that can be related either to increased absorption of water vapor in ageing / dirty sample lines, or unintentional changes in flux filtering following personnel changes.

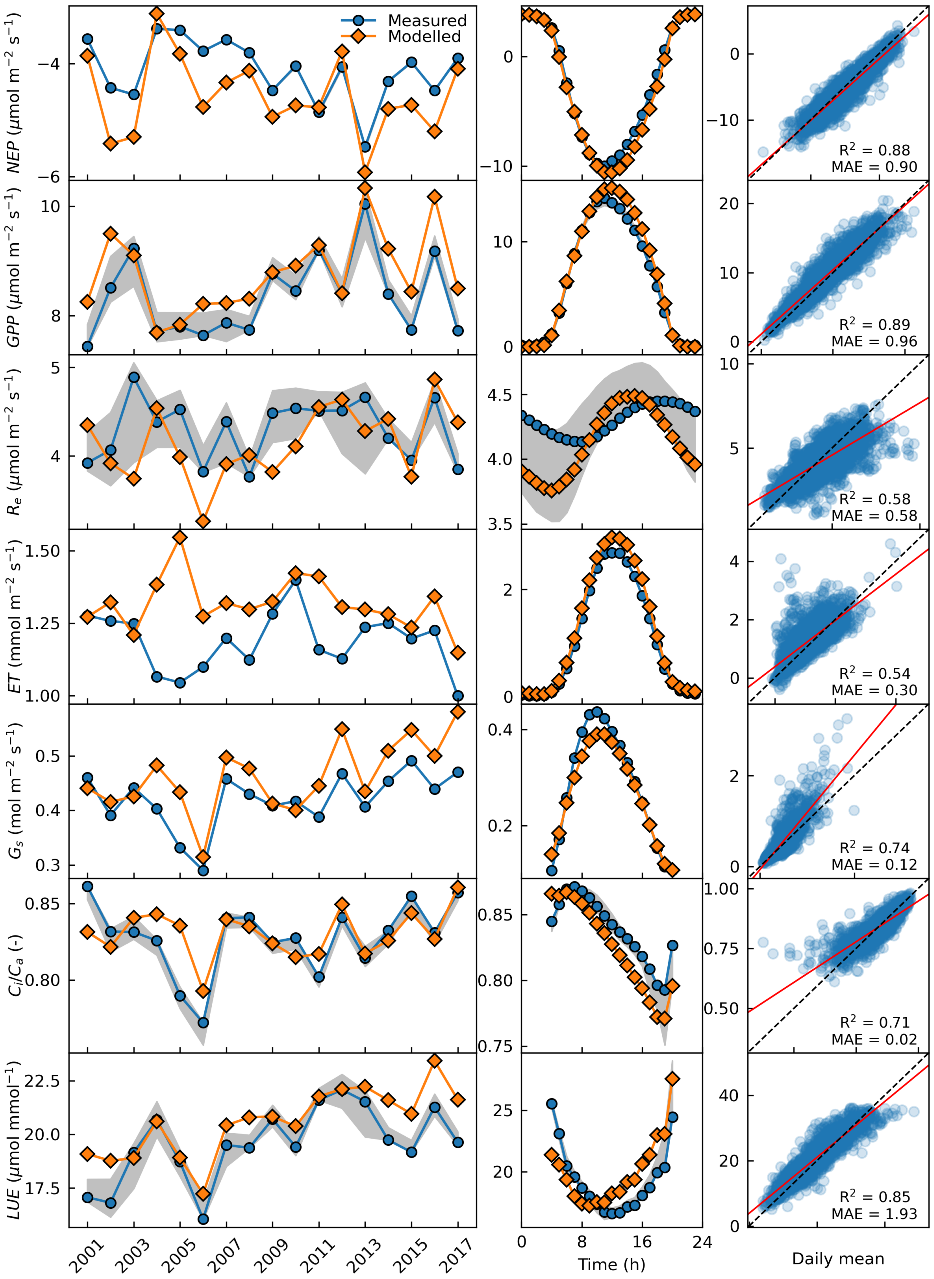

Figure S8: Modeled and measured (night-time T_s partitioning) May-Sept NEP, GPP, R_e, ET, ecosystem surface conductance (G_s), internal to external CO_2 (C_i/C_a) and light-use efficiency (LUE). Left panel shows annual May-Sept means, middle panel median diurnal course and right panel the daily modelled (y-axis) against daily measured (x-axis) means. Gap-filled moments are omitted from comparison. For G_s, C_i/C_a and LUE only data from daytime are included. Shaded area represents range produced by different partitioning methods. In rightmost panels, dashed line represents the 1:1 relation, and MAE and R^2 denote the mean absolute error and the coefficient of determination, respectively.

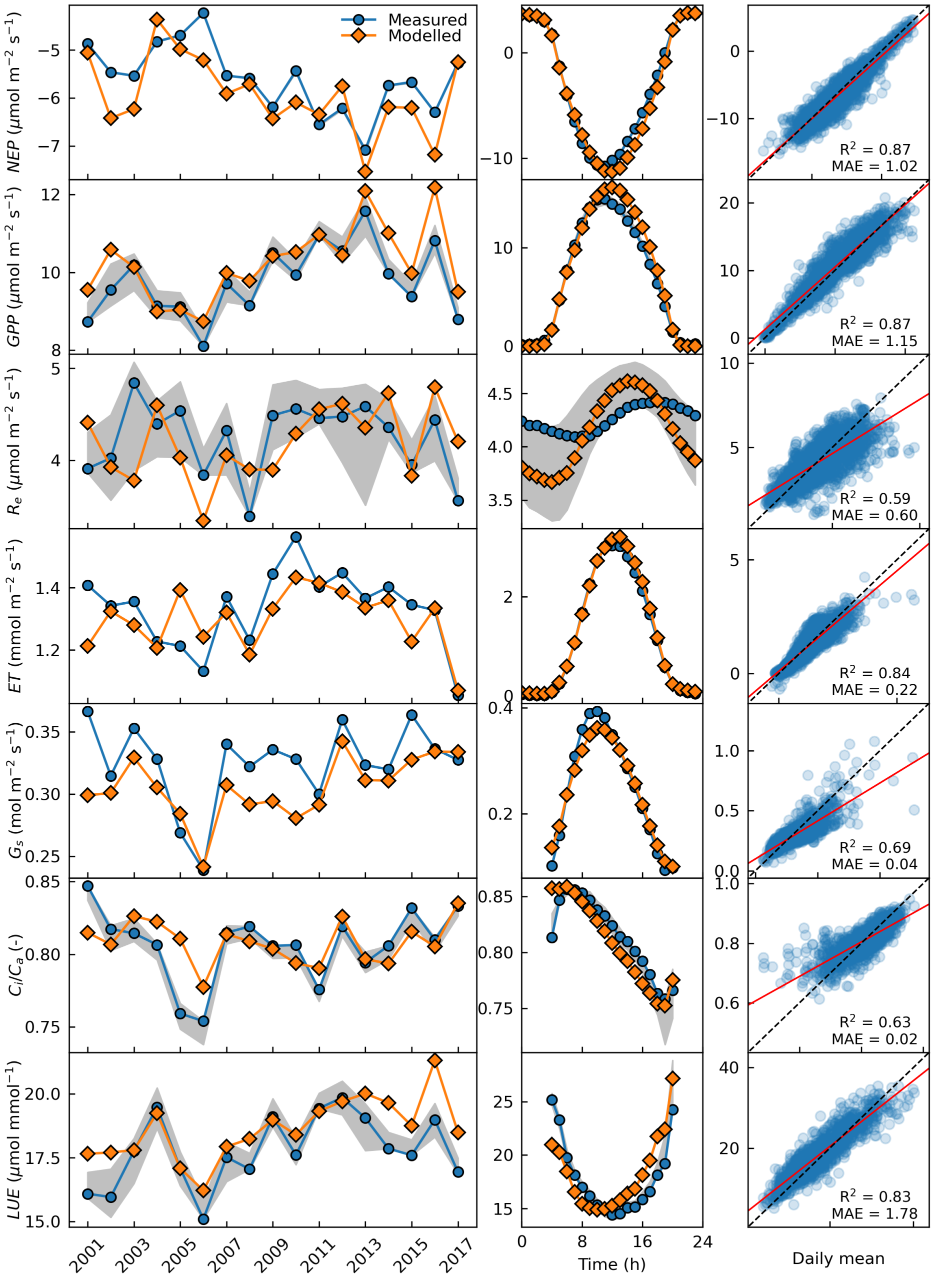

Figure S9: As Fig. S8 but only with dry-canopy conditions included in comparison (i.e. excluding times with rain during past 12 h).

Modeled thinning effect on GPP

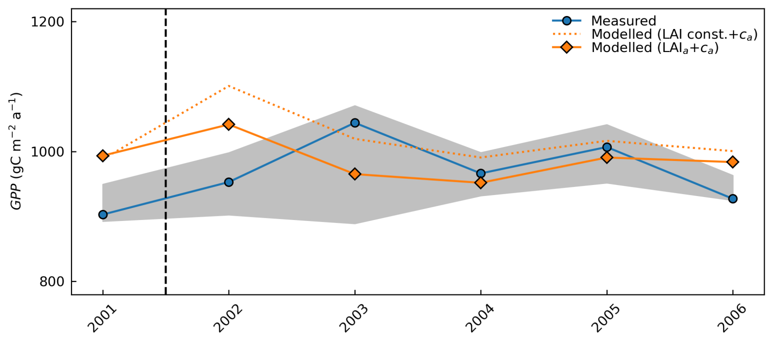

Figure S10: Measured and modelled GPP response to thinning of 25–30% of stand leaf area in 2002 (vertical dashed line). Model results are shown for scenarios accounting for thinning (LAI$_a$+c_a) and no thinning (LAI const.+c_a). The results indicate that the thinning response on GPP was masked by inter-annual variability in meteorological conditions. Shaded area represents observed GPP range by different partitioning methods.

S7 Effect of leaf nitrogen content on GPP trend

Leaf chemistry data (1995–2013) from the ICP Level2 long-term forest monitoring plots in Finland indicate statistically significant increase in Scots pine leaf nitrogen (N_l) content both in 1st and 2nd year needles (Fig. S11), while no trends in other leaf nutrients have been found (Merilä and Jortikka, 2017). The reason for such growth in N_l is unknown as nitrogen deposition has not increased in Finland (Fig. S12). Assuming sensitivity of V_{cmax25} to $N_l \sim 9.7$ μmol g^{-1} N s^{-1} (Kattge et al., 2009), and Scots pine SLA 6.8 m^{2}kg^{-1} (Härkönen et al., 2015), the observed mean N_l trend of 0.23 mg g^{-1} a^{-1} (or ca 1.8 % a^{-1}) would increase V_{cmax25} by ca. 5 μmol m^{-2} s^{-1} (or ca. 10 %) from 2001 to 2017. The impact of such change in V_{cmax25} was introduced into model simulations, the resulting positive trend in GPP (+0.1 % a^{-1}) was roughly 2/3 of the of the direct c_a effect.

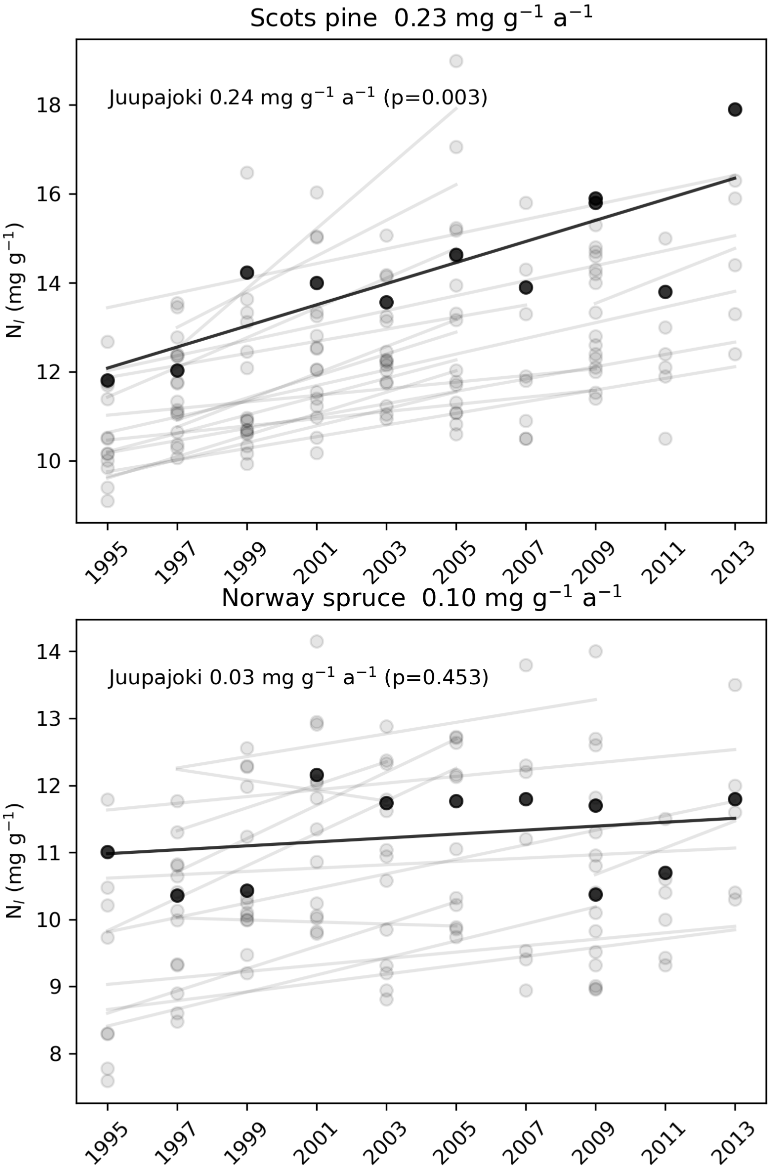

Figure S11: Trends in leaf nitrogen content (N_l, 2nd year needles) of Scots pine and Norway spruce measured across Finland at the ICP Level2 intensive sites. The Juupajoki-site is located close to the SMEAR II site. For Scots pine, 11 of the 15 sites show statistically significant increase in N_l ($p < 0.05$), whereas for spruce the ratio is 4 of 9.

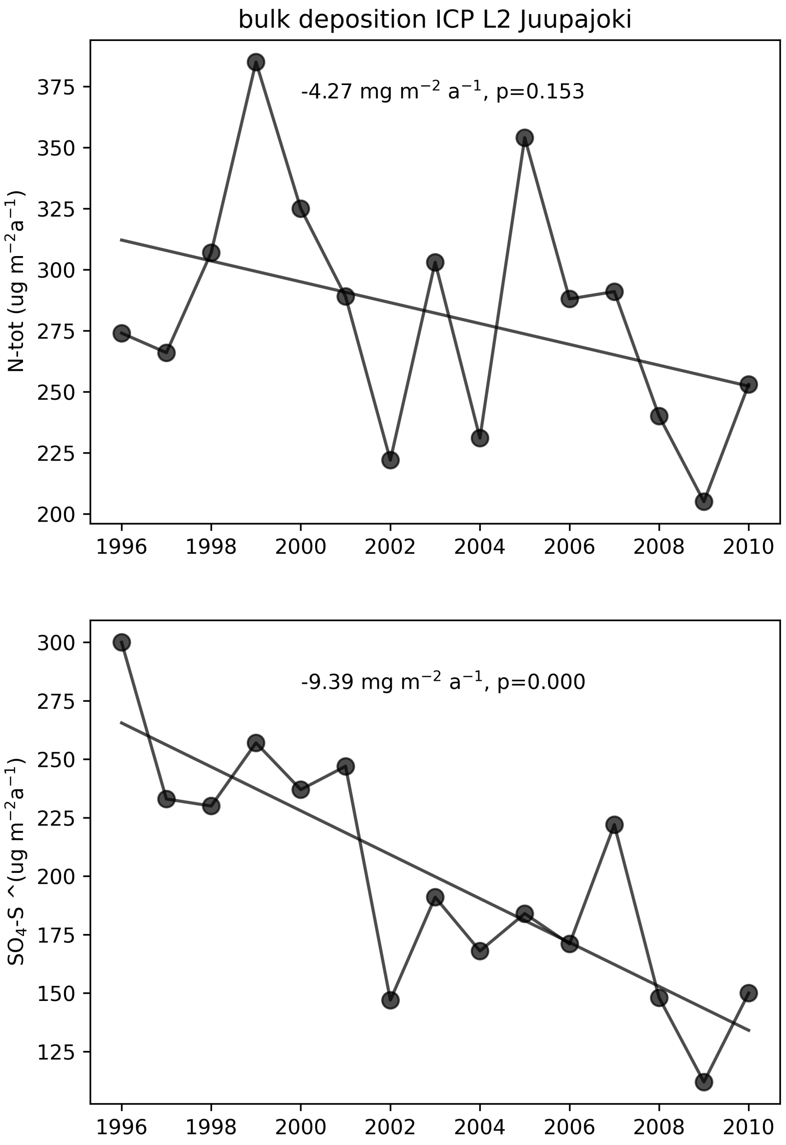

Figure S12: Trends in bulk nitrogen and sulphur as sulphur dioxide (SO_4) deposition at Juupajoki ICP Level2 site, Southern Finland.

S8 Respiration trend in 2001–2017

As APES does not model changes in soil carbon stock, a soil carbon model Yasso07 (Tuomi et al., 2009) was used to explore to what degree heterotrophic respiration (R_h) depends on trend in litter input. In the simulations, the European parameter set (Tuomi et al., 2011) and constant climatic conditions were used. These were based on long-term (1997–2018) averages yielding annual T_a 4.2 °C (amplitude 12.9 °C) and precipitation 669 mm. The soil carbon pools were initialized by 10 000 year spin-up by constant litter input, equalling that in 2001.

For consistency, we assumed annual litter inputs are proportional to leaf mass (from LAI_a; thus representing an upper limit for trends in litter input) as determined by allometric relations and organ-specific turnover rates. We used the following fine root / leaf mass ratios: 0.5 (pine), 0.42 (spruce) and 1.0 (deciduous) (Helmisaari et al., 2007; Johansson, 2007). The total coarse root and branch mass was computed using ratios 9.0 (pine) and 4.5 (spruce, deciduous) (Ilvesniemi et al., 2009). The ground vegetation litter input was set constant, 50 gC m^{-2} a^{-1}, and divided equally between leaf and fine root litter. Turn-over rates were based on Liski et al. (2006). Partial stand thinning in winter 2002 provided additional input of 63 and 52 gC m^{-2} needle/leaf and fine-root litter, respectively, while stumps and harvest residues yielded coarse litter input of 630 gC m^{-2} (Vesala et al., 2005). The respective inputs for snow damages in 2010 were approximated from biomass inventories to be 6, 5, and 7 gC m^{-2} (Fig. S13).

The predictions suggest that thinning in 2001 increased R_h by $<$20% (or $<$50 gC m^{-2}a^{-1}) during the first post-thinning years (Fig. S13). The change in R_h over the whole study period was on the order of 10% (or $<$30 gC m^{-2}a^{-1}). However, when annual weather data was used in the Yasso07 simulations, the resulting inter-annual

variability masked the impact of long-term changes in litter input (not shown). The R_h around 300–330 gC m^{-2}a^{-1} is consistent with Kolari et al. (2009) who showed annual soil respiration varies between 530 and 640 gC m^{-2}a^{-1} at the site, of which R_h contributes ca. 55% (Pumpanen et al., 2015). These agree with 40%–70% reported from other boreal forests (Högberg et al., 2005; Olsson et al., 2005).

Putting these into a perspective for R_e, the results suggest R_a contributes roughly two thirds of R_e and should also dominate the respiration trend. Adopting the common assumption that R_a is proportional to GPP, we can write $R_e = R_a + R_h = (1 - CUE) \times GPP + R_h$, where $CUE = 1 - R_a/GPP$ is ecosystem carbon use efficiency. In boreal forests CUE is shown to range from 0.3 to 0.6 (Goulden et al., 2011; de Lucia et al., 2007; Ryan et al., 1997), and clusters around 0.5 as average for global forests (de Lucia et al., 2007). Specifically for the studied site, Ilvesniemi et al. (2009) estimated CUE between 0.34 and 0.52.

Assuming constant CUE, we can write change in R_e as δR_e = (1 - CUE) $\times$ dGPP + δR_h. Setting CUE to range from 0.3 to 0.6, $\delta GPP \sim$ 130 (Tables 1 & 2) over the 2001-2017 period yields δR_a = 50–90 gC m^{-2}. This corresponds to 2.9–5.3 gC m^{-2} a^{-1}, which is alone equal or several times larger than what was observed for annual R_e (Tables 1 & 2). Adding δR_h < 30 gC m^{-2} (Fig. S13), the trend in R_e would be from 4.5 to 7.0 gC m^{-2} a^{-1}. This is 2–4 times larger than the observed EC-based trend (Table 2) suggesting that CUE may have increased and/or R_h decreased rather than increased during the study period. Fig. S14 shows near-linear decrease of R_e/GPP -ratio during the study period.

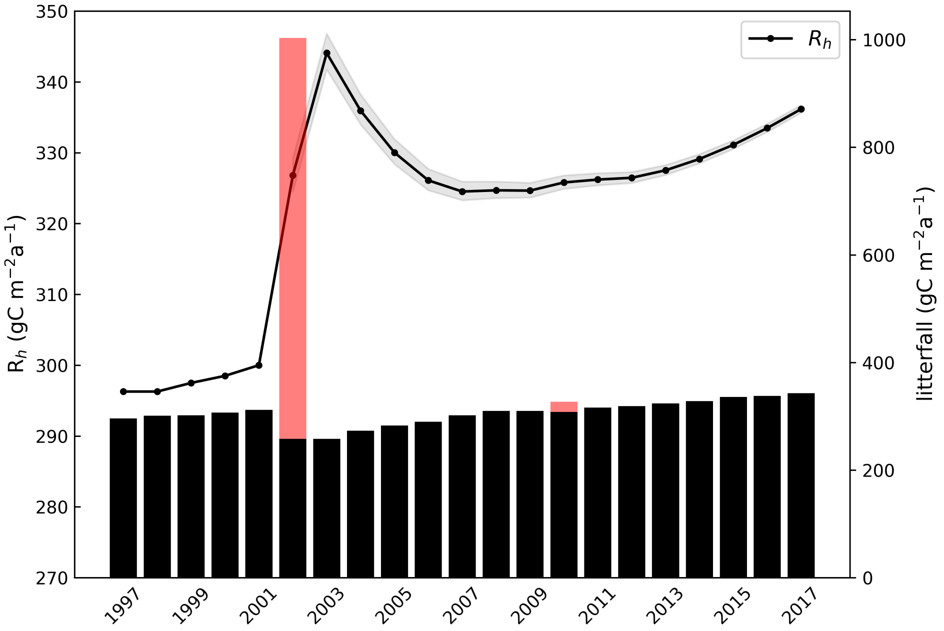

Figure S13: Modeled response of annual heterotrophic respiration (R_h) to litter input as response to LAI growth and thinning in winter 2001/2002.

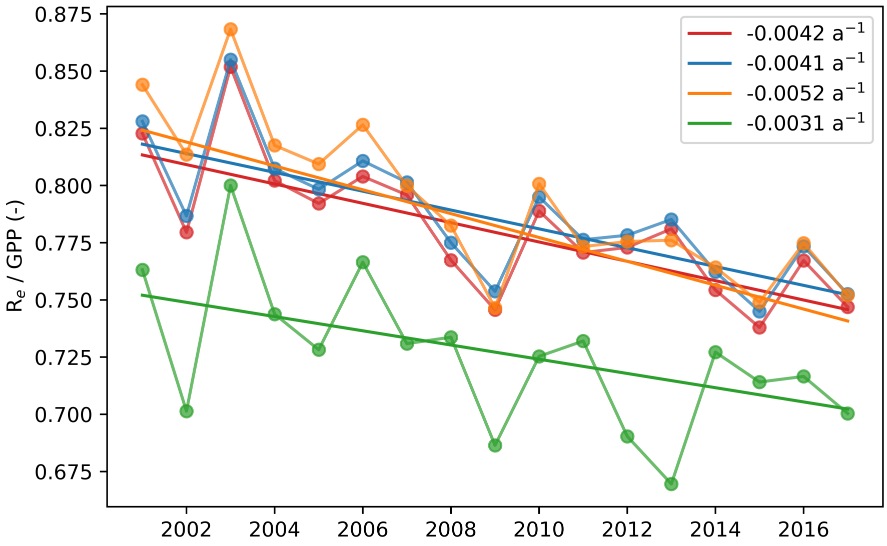

Figure S14: Ratio of annual R_e and GPP. Colors are different flux partitioning methods, see Fig. S1

References

Aubinet, M., Vesala, T., Papale, D., 2012. Eddy covariance: a practical guide to measurement and data analysis. Springer Science & Business Media.

Bruguiére, A., 2020. Lehtialaindeksin ennustaminen laserkeilauksella boreaalisessa metsässä. Bachelor's thesis. University of Eastern Finland, School of Forest Sciences.

Campbell, G.S., Norman, J.M., 1998. Introduction to Environmental Biophysics. Springer. 2nd edition.

Collatz, G., Berry, J., Farquhar, G., Pierce, J., 1990. The relationship between the rubisco reaction mechanism and models of photosynthesis. Plant, Cell & Environment 13, 219–225.

Eugster, W., Senn, W., 1995. A cospectral correction model for measurement of turbulent NO2 flux. Boundary-Layer Meteorology 74, 321–340.

Goulden, M.L., McMillan, A., Winston, G., Rocha, A., Manies, K., Harden, J.W., Bond-Lamberty, B., 2011. Patterns of NPP, GPP, respiration, and NEP during boreal forest succession. Global Change Biology 17, 855–871.

Härkönen, S., Lehtonen, A., Manninen, T., Tuominen, S., Peltoniemi, M., 2015. Estimating forest leaf area index using satellite images: comparison of k-NN based Landsat-NFI LAI with MODIS-RSR based LAI product for Finland. Boreal Environment Research 20, 181–195.

Helmisaari, H.S., Derome, J., Nöjd, P., Kukkola, M., 2007. Fine root biomass in re-

lation to site and stand characteristics in norway spruce and scots pine stands. Tree Physiology 27, 1493–1504.

Högberg, P., Nordgren, A., Högberg, M., Ottosson-Löfvenius, M., Olsson, P., Linder, S., et al., 2005. Fractional contributions by autotrophic and heterotrophic respiration to soil-surface CO2 efflux in boreal forests. SEB experimental biology series , 251–267.

Ilvesniemi, H., Levula, J., Ojansuu, R., Kolari, P., Kulmala, L., Pumpanen, J., Launiainen, S., Vesala, T., Nikinmaa, E., 2009. Long-term measurements of the carbon balance of a boreal Scots pine dominated forest ecosystem. Boreal Environment Research 14, 731–753.

Johansson, T., 2007. Biomass production and allometric above-and below-ground relations for young birch stands planted at four spacings on abandoned farmland. Forestry 80, 41–52.

Kattge, J., Knorr, W., 2007. Temperature acclimation in a biochemical model of photosynthesis: a reanalysis of data from 36 species. Plant, cell & environment 30, 1176–1190.

Kattge, J., Knorr, W., Raddatz, T., Wirth, C., 2009. Quantifying photosynthetic capacity and its relationship to leaf nitrogen content for global-scale terrestrial biosphere models. Global Change Biology 15, 976–991.

Katul, G.G., Mahrt, L., Poggi, D., Sanz, C., 2004. One-and two-equation models for canopy turbulence. Boundary-layer meteorology 113, 81–109.

Keenan, T., Sabate, S., Gracia, C., 2010. Soil water stress and coupled photosynthesis–conductance models: Bridging the gap between conflicting reports on the relative

roles of stomatal, mesophyll conductance and biochemical limitations to photosynthesis. Agricultural and Forest Meteorology 150, 443–453.

Kljun, N., Calanca, P., Rotach, M., Schmid, H.P., 2015. A simple two-dimensional parameterisation for Flux Footprint Prediction (FFP). Geoscientific Model Development 8, 3695.

Kolari, P., Chan, T., Porcar-Castell, A., Bäck, J., Nikinmaa, E., Juurola, E., 2014. Field and controlled environment measurements show strong seasonal acclimation in photosynthesis and respiration potential in boreal scots pine. Frontiers in Plant Science 5, 717.

Kolari, P., Kulmala, L., Pumpanen, J., Launiainen, S., Ilvesniemi, H., Hari, P., Nikinmaa, E., 2009. CO2 exchange and component CO2 fluxes of a boreal Scots pine forest. Boreal Environment Research 14, 761–783.

Lasslop, G., Reichstein, M., Papale, D., Richardson, A.D., Arneth, A., Barr, A., Stoy, P., Wohlfahrt, G., 2010. Separation of net ecosystem exchange into assimilation and respiration using a light response curve approach: critical issues and global evaluation. Global Change Biology 16, 187–208.

Launiainen, S., 2010. Seasonal and inter-annual variability of energy exchange above a boreal Scots pine forest. Biogeosciences 7, 3921–3940.

Launiainen, S., Katul, G.G., Lauren, A., Kolari, P., 2015. Coupling boreal forest CO2, H2O and energy flows by a vertically structured forest canopy – soil model with separate bryophyte layer. Ecological Modelling 312, 385–405.

Leppä, K., Korkiakoski, M., Nieminen, M., Laiho, R., Hotanen, J.P., Kieloaho, A.J.,

Korpela, L., Laurila, T., Lohila, A., Minkkinen, K., et al., 2020. Vegetation controls of water and energy balance of a drained peatland forest: Responses to alternative harvesting practices. Agricultural and Forest Meteorology 295, 108198.

Linderholm, H.W., 2006. Growing season changes in the last century. Agricultural and Forest Meteorology 137, 1–14.

Liski, J., Lehtonen, A., Palosuo, T., Peltoniemi, M., Eggers, T., Muukkonen, P., Mäkipää, R., 2006. Carbon accumulation in finland's forests 1922–2004 – an estimate obtained by combination of forest inventory data with modelling of biomass, litter and soil. Annals of Forest Science 63, 687–697.

de Lucia, E.H., Drake, J.E., Thomas, R.B., Gonzalez-Meler, M., 2007. Forest carbon use efficiency: is respiration a constant fraction of gross primary production? Global Change Biology 13, 1157–1167.

Mammarella, I., Launiainen, S., Gr¨nholm, T., Keronen, P., Pumpanen, J., Rannik, Ü., Vesala, T., 2009. Relative humidity effect on the high-frequency attenuation of water vapor flux measured by a closed-path eddy covariance system. Journal of Atmospheric and Oceanic Technology 26, 1856–1866.

Mammarella, I., Peltola, O., Nordbo, A., Järvi, L., Rannik, Ü., 2016. Quantifying the uncertainty of eddy covariance fluxes due to the use of different software packages and combinations of processing steps in two contrasting ecosystems. Atmospheric Measurement Techniques 9, 4915–4933.

Markkanen, T., Rannik, Ü., Keronen, P., Suni, T., Vesala, T., 2001. Eddy covariance fluxes over a boreal Scots pine forest. Boreal Environment Research 6, 65–78.

Marklund, L.G., 1988. Biomass functions for pine, spruce and birch in Sweden. Rapport-Sveriges Lantbruksuniversitet, Institutionen för Skogstaxering (Sweden) .

Medlyn, B., Dreyer, E., Ellsworth, D., Forstreuter, M., Harley, P., Kirschbaum, M., Le Roux, X., Montpied, P., Strassemeyer, J., Walcroft, A., et al., 2002. Temperature response of parameters of a biochemically based model of photosynthesis. II. a review of experimental data. Plant, Cell & Environment 25, 1167–1179.

Merilä, P., Jortikka, S., 2017. Foliar chemistry on the intensive monitoring plots. Forest Condition Monitoring in Finland – National Report. 27, 1421–1434.

Nordbo, A., Kekäläinen, P., Siivola, E., Mammarella, I., Timonen, J., Vesala, T., 2014. Sorption-caused attenuation and delay of water vapor signals in eddy-covariance sampling tubes and filters. Journal of Atmospheric and Oceanic Technology 31, 2629–2649.

Nordbo, A., Launiainen, S., Mammarella, I., Lepparanta, M., Huotari, J., Ojala, A., Vesala, T., 2011. Long-term energy flux measurements and energy balance over a small boreal lake using eddy covariance technique. Journal of Geophysical Research: Atmospheres 116.

Olsson, P., Linder, S., Giesler, R., Högberg, P., 2005. Fertilization of boreal forest reduces both autotrophic and heterotrophic soil respiration. Global Change Biology 11, 1745–1753.

Pumpanen, J., Kulmala, L., Lindén, A., Kolari, P., Nikinmaa, E., Hari, P., 2015. Seasonal dynamics of autotrophic respiration in boreal forest soil estimated by continuous chamber measurements. Boreal Environmental Research 20, 637–650.

Rannik, Ü., Vesala, T., 1999. Autoregressive filtering versus linear detrending in estimation of fluxes by the eddy covariance method. Boundary-Layer Meteorology 91, 259–280.

Reichstein, M., Falge, E., Baldocchi, D., Papale, D., Aubinet, M., Berbigier, P., Bernhofer, C., Buchmann, N., Gilmanov, T., Granier, A., et al., 2005. On the separation of net ecosystem exchange into assimilation and ecosystem respiration: review and improved algorithm. Global Change Biology 11, 1424–1439.

Repola, J., et al., 2009. Biomass equations for Scots pine and Norway spruce in Finland. Silva Fennica 43, 625–647.

Ryan, M.G., Lavigne, M.B., Gower, S.T., 1997. Annual carbon cost of autotrophic respiration in boreal forest ecosystems in relation to species and climate. Journal of Geophysical Research: Atmospheres 102, 28871–28883.

Tahvanainen, T., Forss, E., 2008. Individual tree models for the crown biomass distribution of scots pine, norway spruce and birch in finland. Forest Ecology and Management 255, 455–467.

Tuomi, M., Rasinmäki, J., Repo, A., Vanhala, P., Liski, J., 2011. Soil carbon model Yasso07 graphical user interface. Environmental Modelling & Software 26, 1358–1362.

Tuomi, M., Thum, T., Järvinen, H., Fronzek, S., Berg, B., Harmon, M., Trofymow, J., Sevanto, S., Liski, J., 2009. Leaf litter decomposition—estimates of global variability based on Yasso07 model. Ecological Modelling 220, 3362–3371.

Vesala, T., Suni, T., Rannik, Ü., Keronen, P., Markkanen, T., Sevanto, S., Grönholm,

T., Smolander, S., Kulmala, M., Ilvesniemi, H., et al., 2005. Effect of thinning on surface fluxes in a boreal forest. Global Biogeochemical Cycles 19.

Watanabe, T., Mizutani, K., 1996. Model study on micrometeorological aspects of rainfall interception over an evergreen broad-leaved forest. Agricultural and Forest Meteorology 80, 195–214.

Wutzler, T., Lucas-Moffat, A., Migliavacca, M., Knauer, J., Sickel, K., Šigut, L., Menzer, O., Reichstein, M., 2018. Basic and extensible post-processing of eddy covariance flux data with reddyproc. Biogeosciences 15, 5015–5030.

Zhou, S., Duursma, R.A., Medlyn, B.E., Kelly, J.W., Prentice, I.C., 2013. How should we model plant responses to drought? an analysis of stomatal and non-stomatal responses to water stress. Agricultural and Forest Meteorology 182, 204–214.

Zhu, W., Chen, G., Jiang, N., Liu, J., Mou, M., 2013. Estimating carbon flux phenology with satellite-derived land surface phenology and climate drivers for different biomes: A synthesis of Ameriflux observations. PloS one 8.
